# Genomic characterization of the type VI secretion system in extraintestinal pathogenic *Escherichia coli*

**DOI:** 10.1128/spectrum.04033-25

**Published:** 2026-06-25

**Authors:** Julia Ienes-Lima, Sonsiray Álvarez-Narváez, Nicolle Lima Barbieri, Klao Runcharoon, Catherine M. Logue

**Affiliations:** 1 Department of Population Health, College of Veterinary Medicine, University of Georgia 1355, Athens, Georgia, USA; 2 Department of Infectious Diseases, College of Veterinary Medicine, University of Georgia 1355, Athens, Georgia, USA; Yan'an University, Yan'an, Shaanxi, China

**Keywords:** extraintestinal pathogenic *E. coli*, ExPEC, type VI secretion system, T6SS, genomic analysis, pathogenesis

## Abstract

Extraintestinal pathogenic *Escherichia coli* (ExPEC) is a distinctive group of *Escherichia coli* strains capable of causing infections outside the gastrointestinal tract of humans and certain animals. ExPEC includes the pathotypes avian pathogenic *E. coli* (APEC), neonatal meningitis *E. coli* (NMEC), sepsis-associated *E. coli* (SEPEC), and uropathogenic *E. coli* (UPEC). These pathotypes share multiple virulence factors, including the Type VI Secretion System (T6SS), a nanomachinery that has been described as important for *E. coli* pathogenesis. In this study, we applied genome-wide association analysis to evaluate the prevalence and evolution of T6SS across 399 ExPEC genomes, including new NMEC (*n* = 78) and APEC (*n* = 68) sequences (*n* = 146). Our findings suggest that among ExPEC, T6SS is more prevalent in NMEC and is more conserved between NMEC and APEC in terms of loci organization and gene content, whereas greater structural variation was observed in SEPEC and UPEC. Additionally, our findings indicate an inverse relationship between T6SS presence and antimicrobial resistance, suggesting that harboring both may incur a fitness cost. Our study provides new insights into the ecological and evolutionary pressures shaping the distribution of T6SS among ExPEC lineages and supports its relevance for further *in vitro* investigation regarding its expression and function in *Escherichia coli*.

**IMPORTANCE:**

Extraintestinal pathogenic *Escherichia coli* (ExPEC) is responsible for severe infections in humans and animals. However, the distribution of the Type VI Secretion System (T6SS) among these pathotypes remains unclear. The T6SS has been implicated in *E. coli* pathogenicity, but its prevalence and structural diversity across ExPEC are still poorly understood due to the lack of genomes available from some pathotypes, especially neonatal meningitis *E. coli* (NMEC) strains. This study introduces new NMEC and avian pathogenic *E. coli* (APEC) genomes among a total of 399 ExPEC genomes analyzed, including sepsis-associated *E. coli* (SEPEC) and uropathogenic *E. coli* (UPEC). Our findings indicate the T6SS as a conserved and predominant virulence factor in NMEC compared with other pathotypes. Moreover, an inverse association between T6SS presence and antimicrobial resistance suggests a potential fitness trade-off that may influence ExPEC evolution. These findings advance our understanding of T6SS in ExPEC and identify this secretion system as a relevant target for future functional and mechanistic studies.

## INTRODUCTION

Type VI Secretion Systems (T6SSs) are transport mechanisms for toxins and effectors and are present in around 25% of Gram-negative bacteria, including ESKAPE pathogens, such as *Klebsiella pneumoniae, Acinetobacter baumanii,* and *Pseudomonas aeruginosa* (1–5). T6SSs were first described in *Vibrio cholerae* V52, which belongs to serogroup O37 in 2006 (6). In the same year, the T6SSs were also found in *Pseudomonas aeruginosa* (7), and since then in many other species, including *E. coli* (8–14).

The structure of the T6SS is evolutionarily related to the cell-puncturing device of T4 bacteriophage (15), and it is composed of a tail complex that includes the inner tube, the spike, and the surrounding sheath, a membrane complex, and a baseplate complex (2, 15–18). In Proteobacteria, these complexes are formed by a core of 13 conserved proteins organized in the *tssA-M* operon and also includes genes *hcp* and *vgrG* (19, 20). The membrane complex, which is specific to the T6SS, forms first from TssJ, TssL, and TssM proteins (21). The membrane complex formation triggers the recruitment of baseplate proteins TssK, TssF, TssG, and TssE and the assembly of the baseplate complex around the VgrG-PAAR complex, followed by polymerization of the inner tube by Hcp proteins (2, 15). Proline-alanine-alanine-arginine (PAAR) repeat proteins are responsible for shaping the tip of the spike through the formation of a conical structure on the VgrG spike (22). Finally, the contractile sheath, made by TssB and TssC proteins, begins to assemble guided and stabilized by TssA (19, 20).

The T6SS can be classified into types I, II, III, and IV based on gene cluster structure and core component evolutionary relationship (23–25). The classification is also associated with the bacterial species, with T6SS^I^ being primarily found in *Proteobacteria*, the T6SS^II^ and T6SS^III^ found exclusively on *Francisella* pathogenicity islands and *Bacteroidetes*, respectively, and T6SS^IV^ found in *Amoebophilus* (23, 26, 27). The T6SS^I^ is subclassified into six subtypes: i1, i2, i3, i4a, i4b, and i5 (28). In *E. coli*, the subtypes T6SS^i2^, T6SS^i1^, and T6SS^i4b^ have been observed (8, 10), and they are usually referred to as T6SS-1, T6SS-2, and T6SS-3, respectively. There is no agreement in the nomenclature used to describe the T6SS subtypes (10), but for the purpose of this manuscript, the term *clusters* will be used to refer to T6SS-1, T6SS-2, and T6SS-3, following the SecReT6 database and previously described studies (9, 19, 25).

Most of the T6SS research in *E. coli* species has been carried out in enteroaggregative *E. coli* (EAEC) and a few extraintestinal pathogenic *E. coli* (ExPEC) (9, 18, 29). Among the ExPEC pathotypes, T6SS has been described as an important virulence factor in avian pathogenic *E. coli* (APEC) pathogenesis (29)*,* in the uropathogenic *E. coli* (UPEC) antibacterial activity (30), and in the binding and invasion of human brain microvascular endothelial cells (HBMEC) in neonatal meningitis *E. coli* (NMEC) (14). Yet, their role and prevalence in sepsis-associated *E. coli* (SEPEC) have not been well described *in vitro* so far.

Currently, *E. coli* strains are classified into 181 O antigens, 53 H antigens, 80 K antigens, and eight phylogenetic groups (A, B1, B2, C, D, E, F, and G) (31, 32). ExPEC isolates usually belong to *E. coli* phylogroups B2 and D, while commensal and InPEC isolates are commonly associated with A and B1 (33–35). In this study, we aimed to evaluate the presence and genomic evolution of the T6SS in extraintestinal pathogenic *E. coli* by genome-wide association analysis. Our goal was to identify the prevalence of T6SS in different ExPEC pathotypes and to determine if there is an association between strain pathogenicity and T6SS presence.

## RESULTS

### Genomic classification of the ExPEC strains

**Fig 1:**
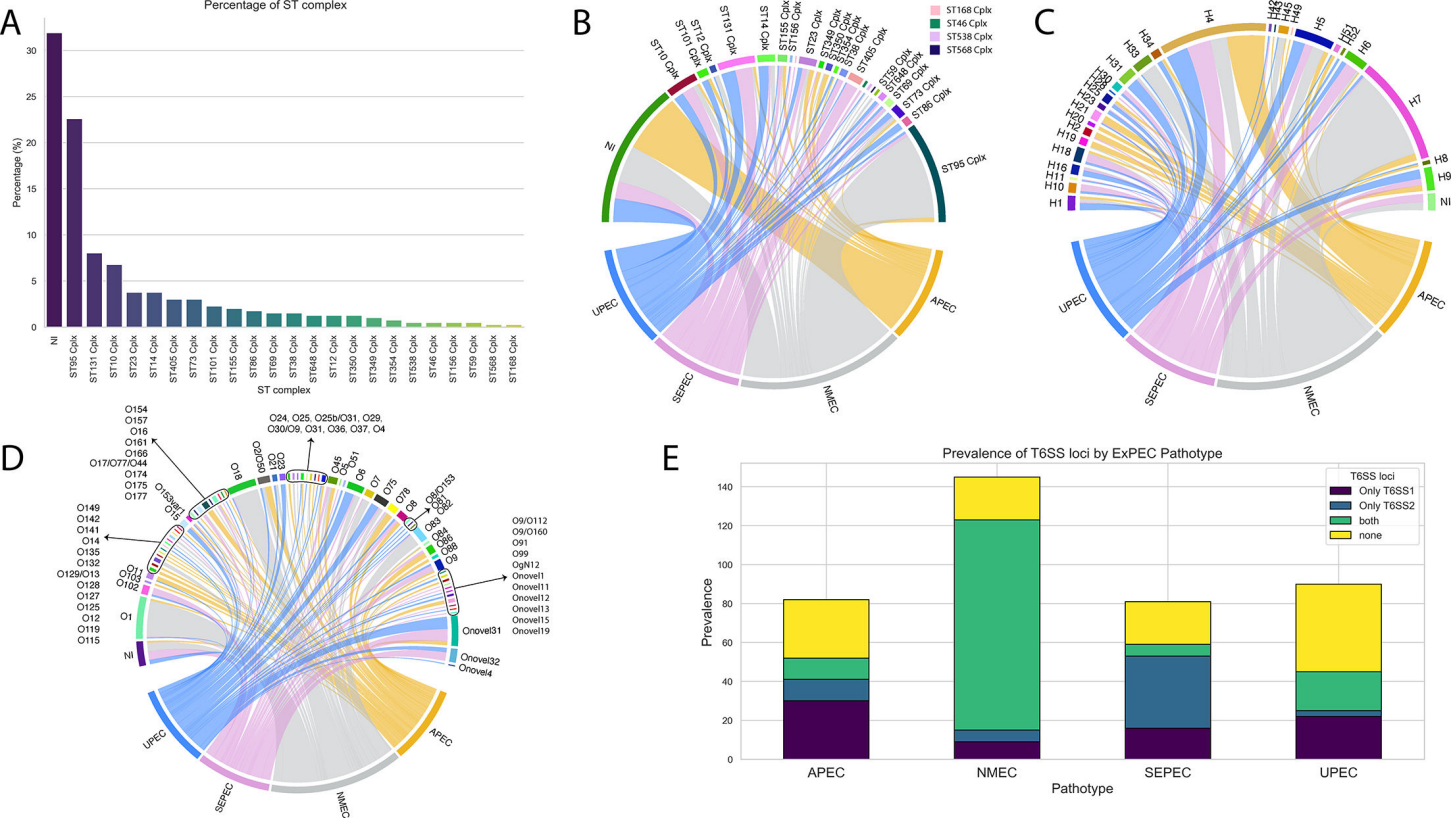
Genome characterization of ExPEC and prevalence of T6SS among the pathotypes. (**A**) Percent prevalence of ST clonal complex among the ExPEC genomes; (**B**) diversity of each ST clonal complex by pathotype (APEC, NMEC, SEPEC, UPEC); (**C**) diversity of H-antigen by pathotype; (**D**) diversity of O-antigen by pathotype; (**E**) prevalence of each T6SS locus among the ExPEC genomes (*N* = 399). NI, not identified.

Among the ExPEC genomes analyzed (*n* = 399), 90 sequence types (STs) were identified, belonging to 23 different ST complexes (Cplx), with the ST95 Cplx being the most prevalent (*n* = 90; 22.7%), followed by ST131 Cplx (*n* = 32; 8%) and ST10 Cplx (*n* = 27; 6.8%). One hundred and twenty-seven genomes (32%) could not be assigned to any ST and were classified as “not identified” (Fig. 1A). ST131 and ST10 Cplx were most common among the SEPEC (15/81, 18.5%; 11/81, 13.6% respectively) and UPEC (13/90, 14.4%; 10/90, 11.1% respectively) genomes, while the ST95 Cplx was predominantly more prevalent among the NMEC (81/145; 56.6%) genomes (Fig. 1B). A total of 74 O-antigens and 29 H-antigens were identified among the ExPEC genomes. H4 was the most prevalent H-type among the ExPEC genomes (*n* = 88; 22%), followed by H7 (*n* = 83; 20.8%), H5 (*n* = 32; 8%), and H9 (*n* = 19; 4.8%). H4 and H5 were commonly observed across all four pathotypes, whereas H7 was most often associated with NMEC strains (Fig. 1C). The most common O-type was O1 (*n* = 46; 11.5%), followed by Onovel31 (*n* = 33; 8.27%) and O18 (*n* = 31; 7.8%) (Fig. 1D), while 30 O-antigens were detected in only one strain each.

### Association between T6SS and ExPEC pathotype

Among the 399 ExPEC genomes, T6SS-1 and T6SS-2 clusters were identified in 151 (38%) genomes; at least one T6SS cluster was observed in 135 (34%) genomes, and 113 (28%) genomes did not harbor any T6SS cluster (Fig. 1E). The T6SS-3 cluster was not identified in any genome analyzed. T6SSs were most prevalent in the NMEC pathotype (122/145; 85%), where most of the strains carried both T6SS-1 and T6SS-2 clusters (110/145; 76%). The majority of SEPEC genomes carried a T6SS-2 (47/81; 58%), while in APEC, the strains primarily carried T6SS-1 (42/83; 51%). The UPEC pathotype had the lowest prevalence of T6SS (47/90; 48%), with most genomes carrying only a T6SS-1 (24/90; 27%), only a T6SS-2 (3/90; 3%), or both T6SS-1 and T6SS-2 clusters (20/90; 22%).

**Fig 2:**
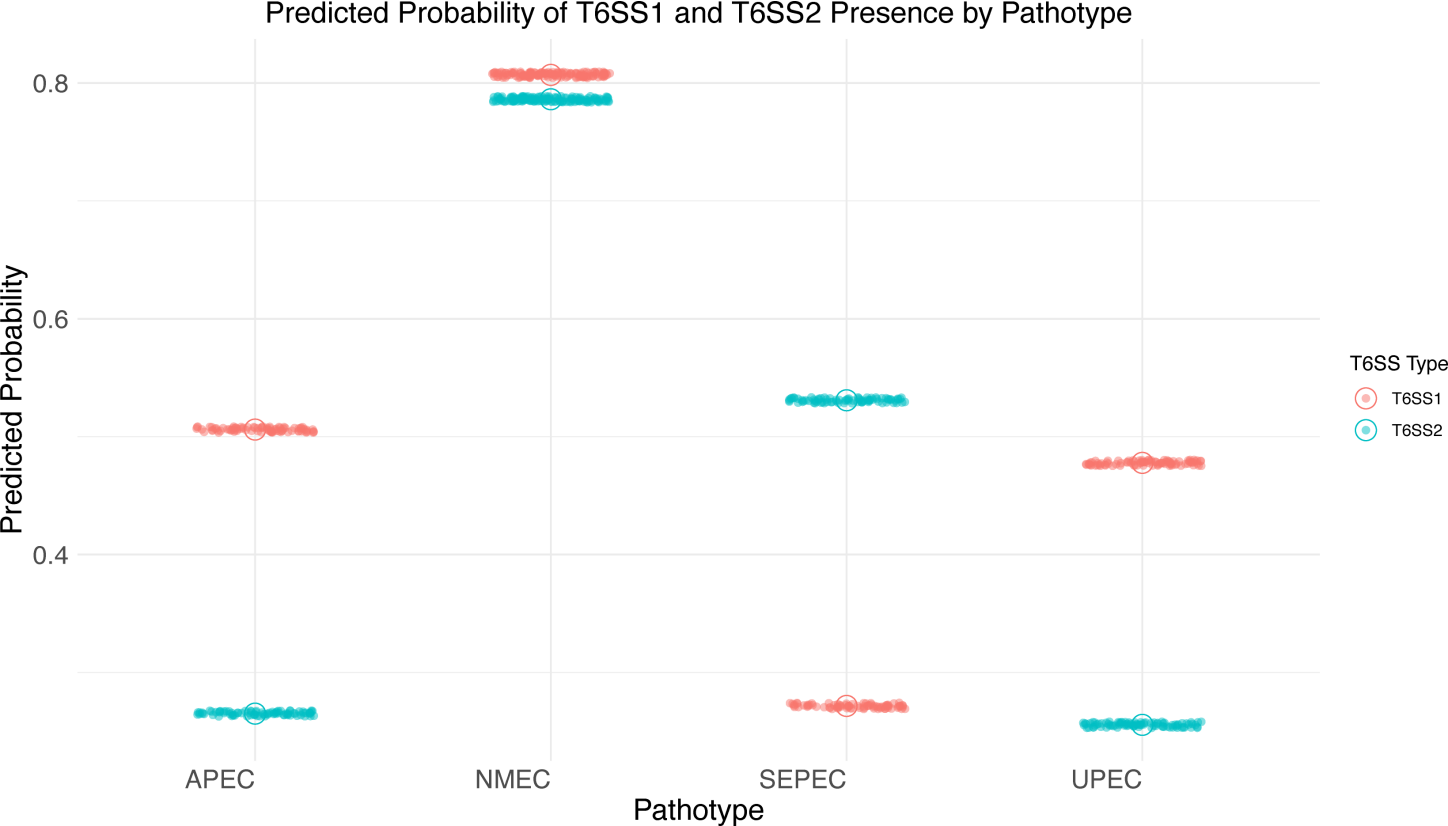
Predicted probability of T6SS-1 and T6SS-2 presence by ExPEC pathotype.

Significant differences were found in the odds ratio (OR) for the presence of T6SS-1 and T6SS-2 among the various ExPEC pathotypes (Table S2). NMEC had the highest estimated probability of harboring both clusters compared with the APEC, SEPEC, and UPEC types (*P* < 0.0001; Fig. 2). The estimated probability of T6SS-1 presence was higher in APEC (estimated difference = 1.01, 95% CI = [0.16, 1.87], *z* = 3.04, *P* = 0.01) and UPEC (estimated difference = 0.90, 95% CI [0.06, 1.74], *z* = 2.75, *P* = 0.03) compared with SEPEC, which have the opposite likelihood for harboring the T6SS-2 cluster compared with both of these pathotypes (*P* = 0.003; *P* = 001; Table S2 and Fig. 2). Additionally, phylogenetic groups B2, D, and G have the highest estimated probability of carrying a T6SS (Fig. S1), as do sequence types ST12, ST14, ST538, ST95 (Fig. S2), and serogroups O18, O2, O83, O75, H5, and H7 (Table S3).

### Structure and location of T6SS in ExPEC

**Fig 3:**
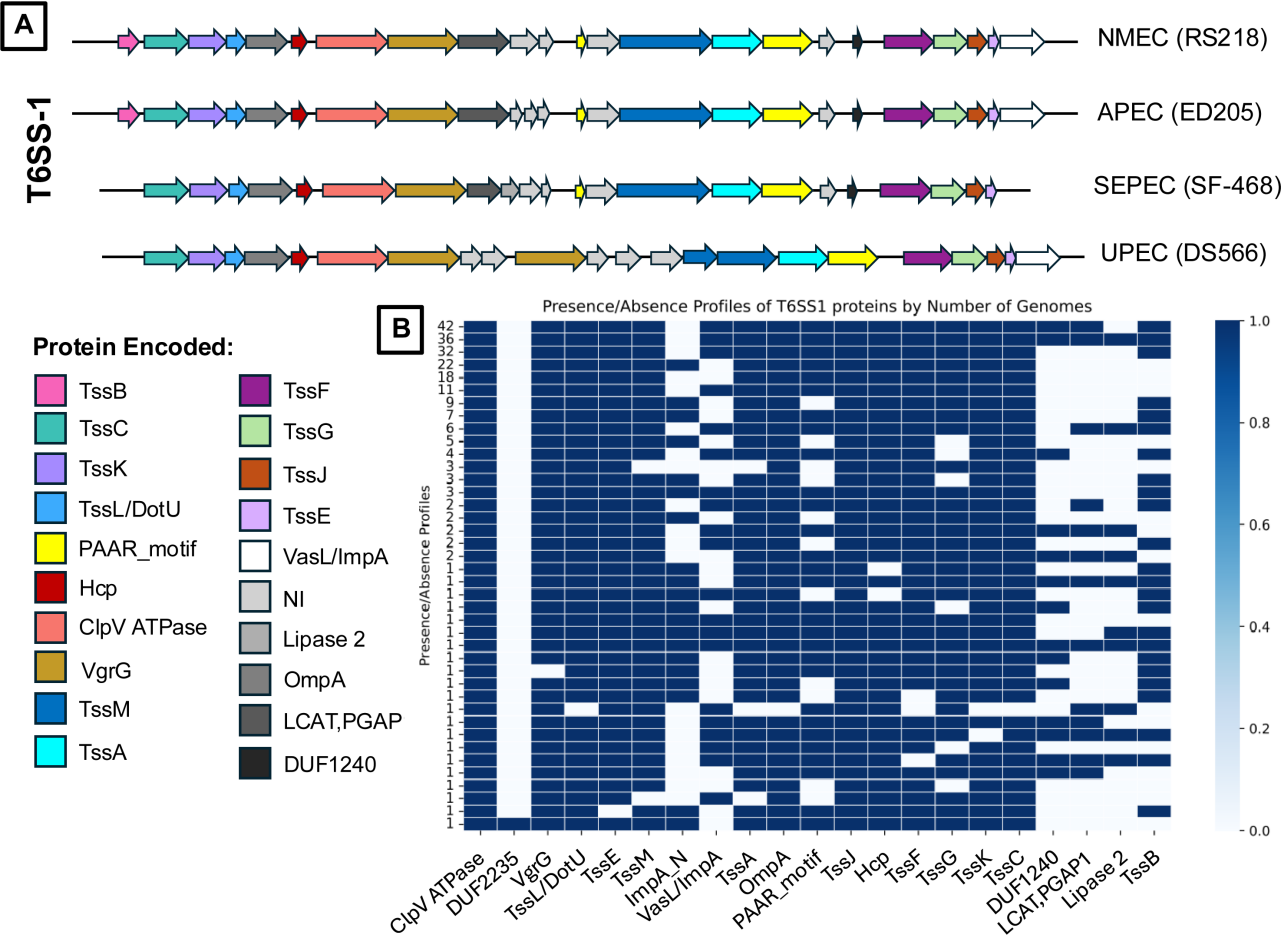
Structure and protein file of T6SS-1 cluster in ExPEC. (**A**) Arrow scheme of T6SS-1 cluster in UPEC, SEPEC, NMEC, and APEC. Each arrow represents a CDS region identified with Prodigal and labeled based on Pfam identification provided by EggNOG. Colored arrows indicate the core proteins, including the Hcp, VgrG, and PAAR proteins, while gray arrows indicate accessory proteins. (**B**) Different structures of the T6SS-1 cluster found in our ExPEC genome collection. The numbers in each row (left) represent the number of sequences that presented their respective cluster structure. Navy blue/1, present; White/0, absent; NI, not identified.

All T6SS identified in this study belong to T6SS-1 and T6SS-2 clusters, were chromosomally encoded, typically associated with HGT elements (Fig. S3 to S8), and displayed a slightly higher GC content than the genomic average of 50.7% (Table 1). Although both clusters shared a conserved set of core structural genes, they differed in gene composition and organization, with T6SS-1 showing a broader diversity and number of accessory proteins and domain arrangements than T6SS-2. These subtype-specific proteins, such as DUF1240 and DUF4123, likely account for the differences in protein-coding sequence content observed between clusters (Fig. S9). The T6SS-1 cluster averaged 25,570 bp, and the reference sequences encompassed 22–24 protein-coding sequences (Fig. 3A).

**TABLE 1:** Nucleotide statistics of T6SS sequences extracted from ExPEC genomes^*a*^

		No. of sequences	Mean GC content	Mean length (bp)	Min. length (bp)	Max. length (bp)	Std dev
T6SS-1							
	APEC	42	56.90%	22,377	20,615	29,754	2,982.9
	NMEC	121	53.60%	27,306	16,818	29,975	3,537.6
	SEPEC	25	57.50%	21,476	15,793	27,879	3,205.6
	UPEC	44	55.70%	31,119	16,260	36,149	5,809.7
	Average		55.93%	25,570	17,372	30,939	3,884
T6SS-2							
	APEC	25	56.30%	19,268	13,887	27,969	5,487.7
	NMEC	111	54.40%	25,150	18,248	29,100	1,178.1
	SEPEC	47	55.80%	15,060	13,215	22,179	3,463.4
	UPEC	23	55.30%	32,333	27,635	43,373	5,296.6
	Average		55.45%	22,953	18,246	30,655	3,856

^*a*^ bp, base pairs.

**Fig 4:**
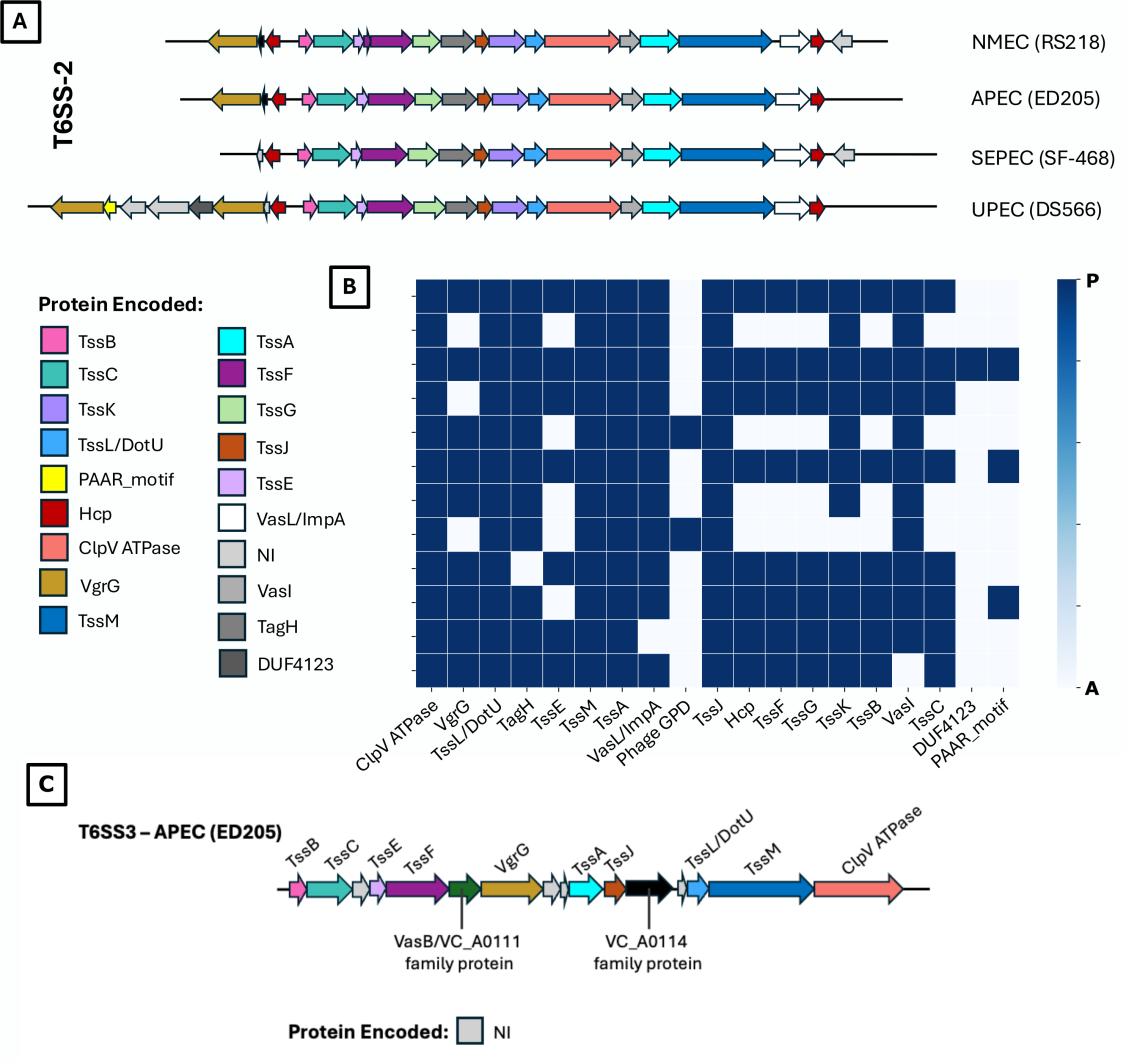
Structure and protein file of T6SS-2 and T6SS-3 clusters in ExPEC. (**A**) Arrow scheme of T6SS-2 cluster in UPEC, SEPEC, NMEC, and APEC. Each arrow represents a CDS region identified with Prodigal and labeled based on Pfam identification provided by EggNOG followed by BLASTp. Colored arrows indicate the core proteins, including the Hcp, VgrG, and PAAR proteins, while gray arrows indicate accessory proteins. (**B**) Different structures of the T6SS-2 cluster found in our ExPEC genome collection. The numbers in each row (left) represent the number of sequences that presented their respective cluster structure. Navy blue/P, present; White/A, absent. (**C**) Arrow scheme of T6SS-3 cluster in APEC, adapted from Ma et al. (9). NI, not identified.

Nonetheless, a total of 40 different protein domain arrangements were identified for T6SS-1 across the ExPEC genomes analyzed, with 31 of these patterns found in five or fewer genomes (Fig. 3B). The Pfam’s associated with genes *clpV* and *tssJ*—based on the nomenclature proposed by Shalom et al. (36)—were present in all T6SS-1 sequences. The T6SS-2 cluster averaged 22,953 bp, and reference sequences encompassed 18–23 protein-coding sequences (Fig. 4A). Only 12 different protein arrangements were identified, with 61% of the clusters (125/206) displaying the most common pattern (Fig. 4B). Five Pfams were observed in all T6SS-2 sequences analyzed, and they were associated with the genes *clpV*, *tssL/dotU*, *tssM*, *tssA*, and *tssJ*. The T6SS-3 cluster (Fig. 4C) was not identified in any ExPEC genome analyzed.

Regarding T6SS completeness and functionally, the screening applied in this study filtered for sequences with a minimum of 52% of query coverage from the reference T6SS sequence. The functionality of T6SS was predicted based on this threshold and on the presence of genes previously described as essential for T6SS functionality, such as *hcp, tagH*, and PAAR protein (11, 12, 22, 37). Among the T6SS-1 sequences, 29/232 sequences do not harbor the PAAR protein, including one out of two that also lacks the *hcp* gene (Fig. 3B). The absence of the PAAR protein was observed in 188/206 sequences of T6SS-2, while 51 and 1 sequences lacked the *hcp* and *tagH* genes, respectively (Fig. 4B). The PAAR protein in T6SS-2 cluster was absent from most SEPEC, APEC, and NMEC isolates, being present only in UPEC isolates, whereas the absence of *hcp* was predominantly associated with SEPEC isolates (Table S4).

### T6SS phylogeny in ExPEC is associated with strain pathotype and phylogroup

**Fig 5:**
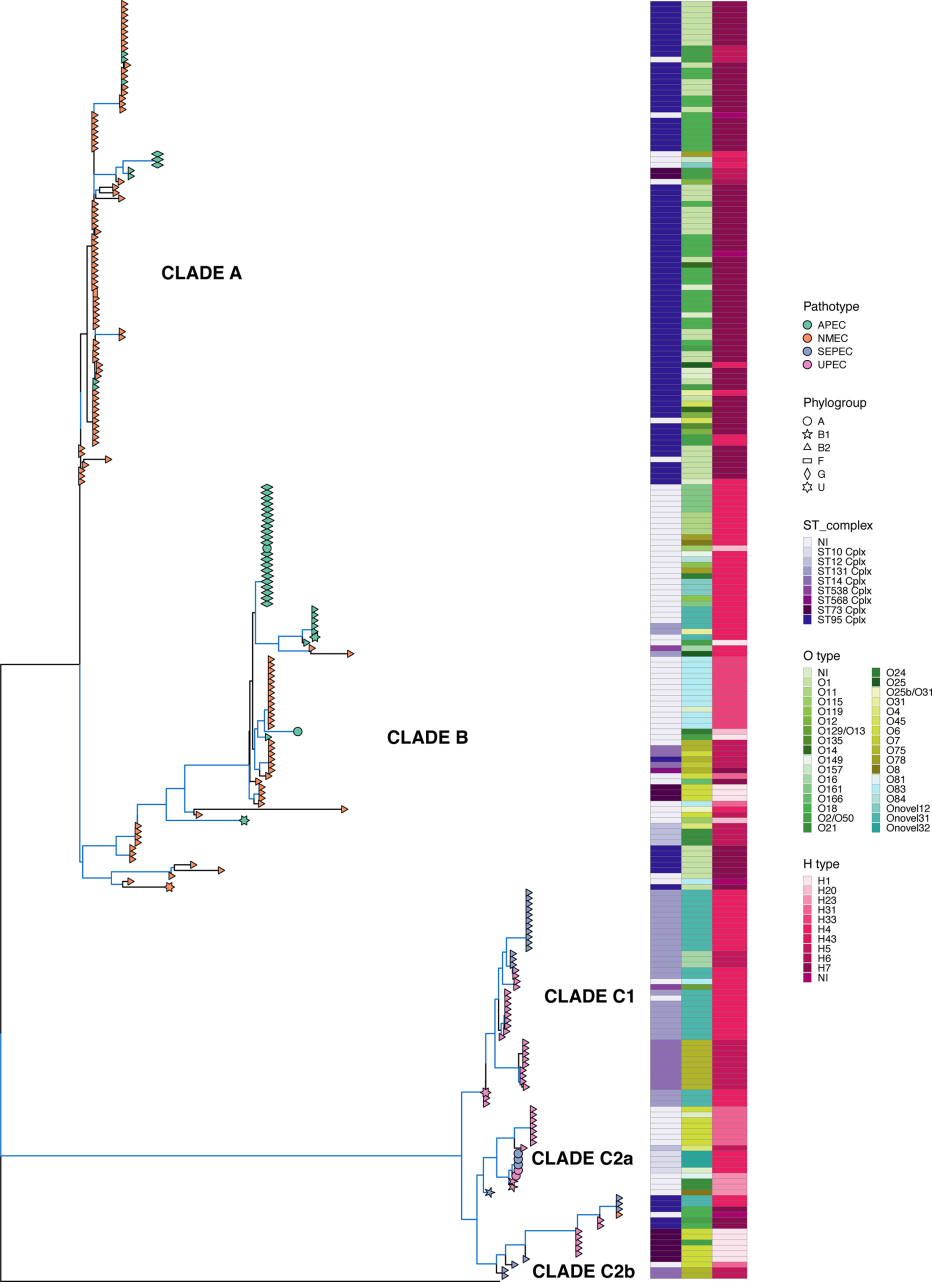
Maximum-likelihood phylogenetic tree of the T6SS-1 cluster rotated to the T6SS sequence of *Vibrio cholerae* strain V52. Branches with bootstrap values higher than 0.9 are highlighted in blue. NI, not identifed.

The phylogenetic analysis of the T6SS-1 sequences classified the ExPEC strains into three major clades (Fig. 5), with the main clade consisting of 87 isolates, primarily NMEC (clade A, bootstrap value = 0.67). Clade B includes most isolates of the APEC and NMEC pathotype, B2 and G phylogroup, and an unidentified ST clonal complex (bootstrap value = 0.96). All SEPEC and UPEC isolates were grouped into clade C, which was subdivided into C1, C2a, and C2b, all with a bootstrap value equal to 1. Clade C1 included mostly isolates Onovel31:H4:ST131Cplx and O75:H5:ST14Cplx, while clade C2a included a few non-identified sequence-types from phylogroups A, B1, and B2. Clade C2b consisted exclusively of strains from phylogroup B1.

**Fig 6:**
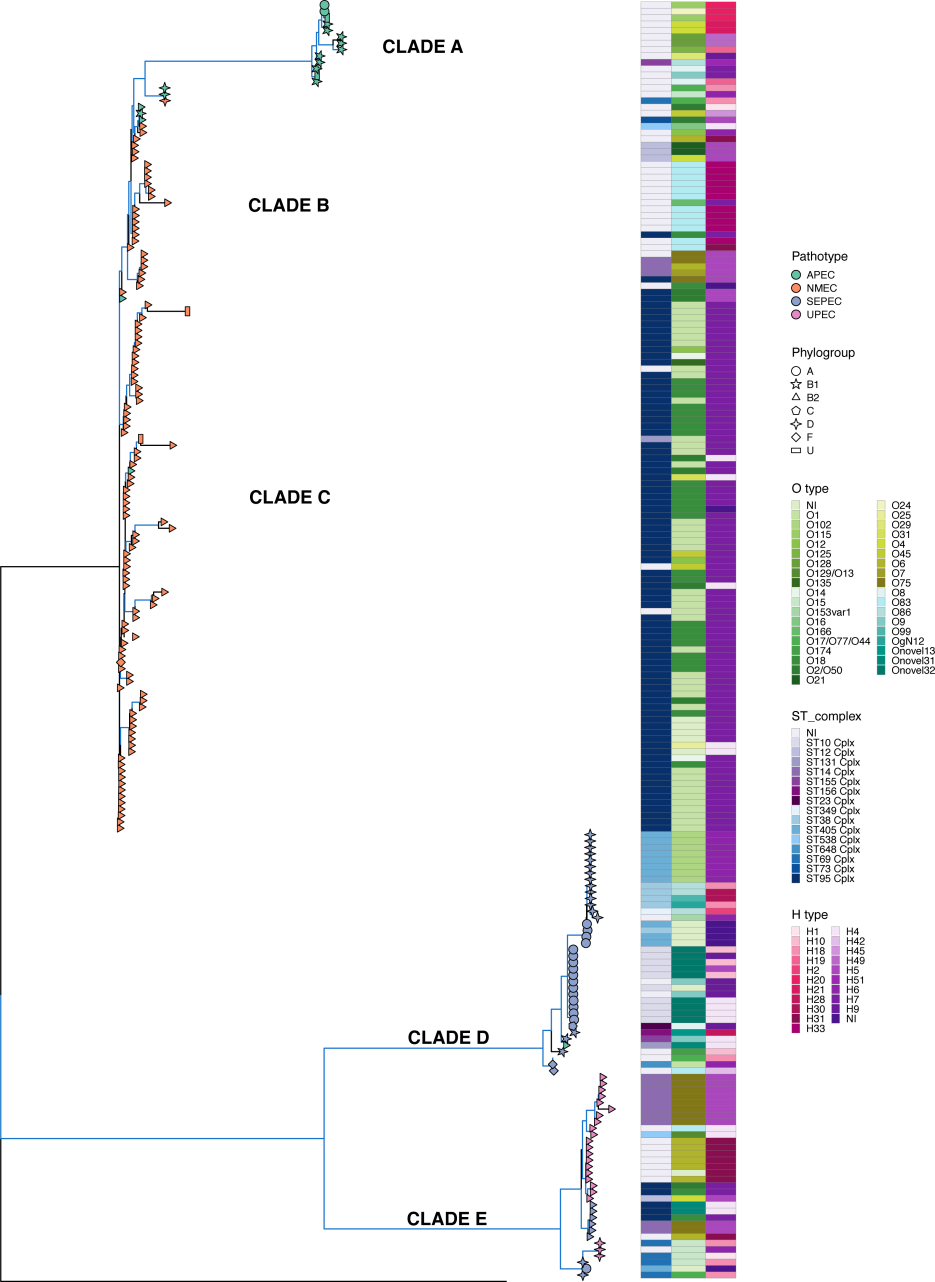
Maximum-likelihood phylogenetic tree of the T6SS-2 cluster rotated to the T6SS sequence of *Vibrio cholerae* strain V52. Branches with bootstrap values higher than 0.9 are highlighted in blue. NI, not identified.

T6SS-2 phylogeny grouped the ExPEC genomes into five clades (Fig. 6), with a major clade consisting of most of the NMEC isolates belonging to the O1:H7 and O18:H7 serotypes and ST95 clonal complex (represented by clade C; bootstrap value = 0.59). Interestingly, clade A comprises only T6SS sequences from APEC and has emerged from clade B, which includes the NMEC strains from unidentified ST complexes and different serotypes, such as O83:H33. Clade D had only SEPEC isolates but from distinct phylogroups (A, B1, D, and F) and ST clonal complexes (ST10 Cplx and ST405 Cplx). On the other hand, clade E consisted of UPEC and a few SEPEC isolates, belonging to the B2 and D phylogroups, and one isolate from phylogroup A (bootstrap value = 1).

The phylogenetic analysis indicates an association between T6SS and NMEC phylogroup and pathotype, which is also supported by the logistic regression analysis previously performed. T6SS-1 from APEC and NMEC shared high similarity, and the same was observed for the T6SS-2 cluster (Fig. S10 and S11). In contrast, T6SS-1 sequences from SEPEC and UPEC shared limited similarity and were highly divergent from those of APEC and NMEC (Fig. S10). Additionally, clades in both phylogenetic trees indicated that T6SS sequences are associated mostly with ExPEC pathotypes and phylogroups than with ST, O, and H types, since they were usually clustered into the same branches in the tree.

### Conflicting relationship between antimicrobial resistance and the presence of T6SS in ExPEC

**Fig 7:**
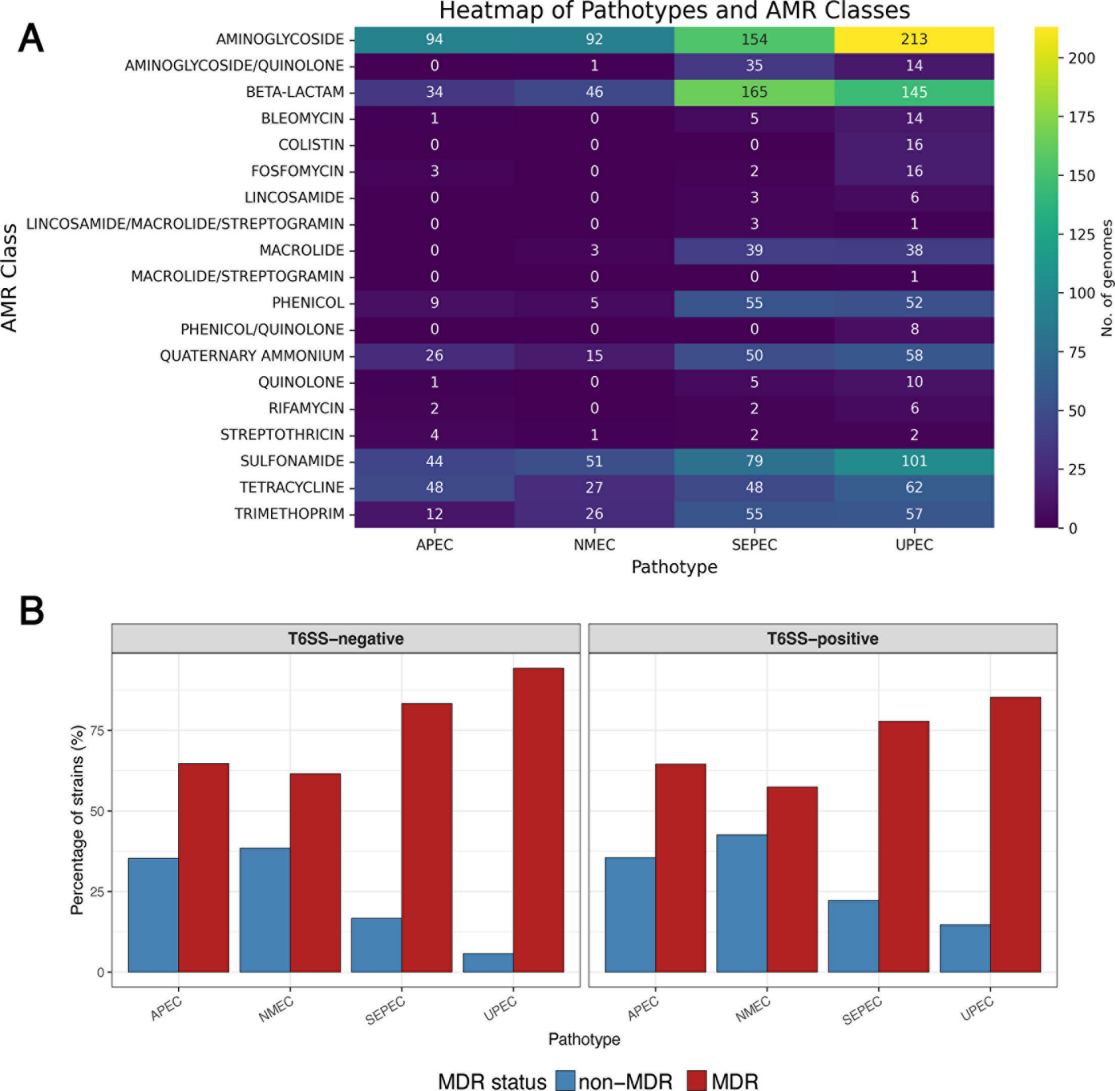
Distribution of AMR classes and multidrug resistance among ExPEC genomes. (**A**) Prevalence of AMR classes among ExPEC genomes. The numbers in each cell indicate the number of genomes harboring each class. (**B**) Comparison of MDR among T6SS-positive (*n* = 173/250, Fisher’s *P* value = 0.019) and T6SS-negative (*n* = 77/250; Fisher’s *P* value = 0.009) strains in which antimicrobial resistance genes were detected (*n* = 250).

A total count of 2,067 antimicrobial resistance genes (AMR) were *in silico* detected among 250 (63%) of the 399 tested ExPEC genomes. The majority of genes were found in UPEC (*n* = 820 genes, average 12 genes/genome) and SEPEC (*n* = 702 genes, average 11 genes/genome), while 278 genes were found in APEC (average six genes/genome), and 267 genes were found in NMEC (average four genes/genome). A diversity of 135 AMR genes was observed, with *bla*_TEM-1_ being the most prevalent (152 counts in general; Fig. S12), especially in UPEC and NMEC strains, although two or more copies of the gene were possible in the same isolate. In APEC and SEPEC, the genes *tetA* and *qacEdelta1* were the most prevalent, respectively. Genes related to resistance to aminoglycosides were observed in all four pathotypes (Fig. 7A). Most UPEC and SEPEC strains also presented a high potential for beta-lactam resistance, while APEC and NMEC strains presented a high prevalence of genes related to tetracycline and sulfonamide resistance, respectively (Fig. 7A).

One hundred and eighty-four strains (46%) were *in silico* predicted to be multidrug resistance (MDR) as their genomes presented resistance to three or more classes of antimicrobials (Fig. S13). The pathotype with the highest number of MDR strains was UPEC (*n* = 62 strains, 69%) which also corresponds to the pathotype with the lowest prevalence of T6SS. On the other hand, only 39 strains (27%) of the NMEC pathotype, with a high prevalence of T6SS, presented MDR (Fig. S14A and B). Statistical analysis identified a positive association between the presence of MDR with UPEC compared with other ExPEC pathotypes (Pearson’s *χ*^2^ = 20.474, df = 3, *P* < 0.001; Fisher’s exact test: *P* < 0.001). Although strains harboring one or both T6SS clusters were also classified as MDR, a significant association between T6SS absence and MDR was observed (Pearson’s *χ*^2^ = 8.178, df = 3, *P* = 0.042; Fisher’s exact test: *P* = 0.045). In addition, a similar MDR-pathotype dynamics was observed in T6SS-negative strains (*n* = 77/250), with UPEC isolates showing a higher MDR prevalence than other pathotypes (Fig. 7B and Table S5).

Association between cluster completeness and MDR was also investigated (Fig. S14C and D). No significant association was observed between MDR and T6SS-1 cluster completeness (Pearson’s *χ*^2^ = 2.086, df = 1, *P* = 0.148; Fisher’s exact test: *P* = 0.154). On the other hand, MDR was significantly associated with T6SS-2 cluster completeness (Pearson’s *χ*^2^ = 9.999, df = 1, *P* = 0.0016; Fisher’s exact test: *P* = 0.0026), with MDR isolates showing a lower frequency of complete T6SS-2 cluster than non-MDR isolates (16.3% vs 34.8%) Altogether, these findings suggest a possible bacterial trade-off between virulence factors, such as the T6SS and antimicrobial resistance genes.

## DISCUSSION

One of the biggest challenges for the *in silico* investigation of T6SS in *E. coli* is the limited availability of genomes from some pathotypes, such as APEC and NMEC. In this study, we included 68 new APEC and 78 new NMEC genomes in our analysis of T6SS prevalence within the ExPEC pathotype, alongside 253 publicly available ExPEC genomes. The present study is the first to evaluate the presence of T6SS in a collection of APEC and NMEC genomes, and its presence in most NMEC B2 strains provides valuable insights regarding its importance for the pathotype pathogenicity.

Our analysis showed that the nucleotide sequence of clusters T6SS-1 and T6SS-2 is not well conserved among ExPEC, but it is still highly prevalent, being present in 70.1% of the genomes analyzed. This finding correlates with the most recent evaluation of T6SS presence across *E. coli* genomes that identified the T6SS in 70.3% of the genomes analyzed; however, no NMEC genomes were included in their analysis (10). Our study observed a difference in the prevalence of specific T6SS clusters between ExPEC pathotypes. A high prevalence of both T6SS-1 and T6SS-2 was only observed in NMEC, which contributes to the hypothesis that T6SS might play an essential role in NMEC pathogenesis, especially in host colonization and biofilm formation, as described in a previous study (38).

The T6SS-1 was found more often in APEC and UPEC strains, while T6SS-2 was the most prevalent cluster among SEPEC strains. These findings are consistent with previous studies that also observed the presence of T6SS-1 in UPEC isolates belonging to phylogroups B2 and D (30, 39), and a higher prevalence of T6SS-1 than T6SS-2 in APEC (9). The absence of T6SS-3 in the analyzed genomes was expected since they have been described mostly in APEC and at a very low prevalence (0.8% among 472 APEC isolates) (9). The same study identified the same cluster structure of APEC T6SS-3 in the enteroaggregative *E. coli* strain EAEC 55989 (9), which has been the only description of this cluster in *E. coli* pathotypes besides APEC. Although the role of T6SS-3 in APEC is still unknown, recent findings suggested a potential function of T6SS-3 in the quorum-sensing mechanism of *Aliivibrio wodanis*, a fish pathogen, allowing the bacteria to co-exist and predominate over *Moritella viscosa,* resulting in the winter ulcer disease in Atlantic salmon (40).

A high similarity between the T6SS-2 of APEC ED205 and NMEC RS218 was reported by Ma et al. (9), a finding consistent with our results, which showed that most T6SS-2 sequences from APEC clustered phylogenetically within the same clade as NMEC T6SS-2 sequences. The T6SS-2 from APEC and NMEC was found to be genetically similar yet distinct from the T6SS-2 of other ExPEC, further supporting the close phylogenetic relationship between APEC and NMEC described in previous studies (41–43). Over the past decade, multiple studies have shown that NMEC and APEC, particularly ST95 strains, share many chromosomal and plasmid-associated virulence factors, highlighting the zoonotic potential of APEC (41–43). NMEC strains also harbor multiple chromosome-carried virulence genes found in UPEC, and both UPEC and NMEC strains generally belong to the B2 phylogenetic group (41). A study based on the sequence-O-type (a combination of O serogroup and the MLST-sequence type) from UPEC and NMEC strains isolated from infants has identified that some strain complexes are exclusively associated with either urosepsis or meningitis, but some complexes were also associated with both infections (44). Based on these findings, Tourret and Denamur (45) proposed that a particular subset of virulence factors and genetic backgrounds could explain the niche specialization observed among these pathotypes (45). In our study, the T6SS clusters were observed in both UPEC and NMEC strains from the B2 phylogroup, suggesting that T6SS is not among the virulence factors associated with niche specialization between these pathotypes.

Recently, a large-scale genomic analysis described the distribution and diversity of T6SS in intestinal and extraintestinal pathogenic *E. coli*, including APEC, SEPEC, and UPEC, but no NMEC genome was included in the study due to the underrepresentation of genomes (10). Our study included 78 and 68 new NMEC and APEC genomes in the genomic analysis, respectively, and phylogenetic analysis showed short branch lengths in clades comprising both APEC and NMEC T6SS sequences, which indicates high genetic similarity at the nucleotide level. A similar finding was previously reported in studies that demonstrated that most amino acid sequences of the OmpA protein from APEC and NMEC strains are 100% identical (42), and no polymorphism between its sequences in both pathotypes was identified (46). All these homologies might contribute to the ability of APEC strains to cause meningitis in a rat model using very similar strategies as NMEC to cross the blood-brain barrier and reach the cerebrospinal fluid, as previously described (42, 46). In addition, a recent study investigated the role of multiple T6SS proteins in adhesion and invasion of HBMEC and found that ImpK and IcmF are involved in cell adherence, while Hcp and ImpK, and IcmF proteins are involved in cell invasion (38), which indicated that T6SS is probably involved in NMEC’s ability to cross the blood-brain barrier.

Interestingly, no direct association between O-antigen or H-type was observed in the phylogenetic analysis. However, an association between the ExPEC pathotype and phylogroup was evident in our results. Our analyses found that ExPEC strains belonging to phylogroups B2, D, and G showed the highest odds for T6SS presence. Taking into consideration the fact that the *E. coli* phylogroups B2 and D comprise most of the ExPEC isolates (47), this finding provides support to the initial hypothesis that T6SS is a conserved structure in ExPEC. The phylogroup G was recently described as an intermediate between phylogenetic groups B2 and F, and it is usually isolated from poultry (31). A previous study observed a prevalence of 43% strains belonging to phylogroup G in a collection of 115 APEC strains (48). This finding aligns with the observations made in this study, as phylogroup G was identified exclusively in APEC strains, and all T6SS sequences from phylogroup G strains were clustered with those from B2 strains of APEC and NMEC. The predicted prevalence of T6SS-1 was higher in phylogroups B2 and G than in the other phylogroups. The predicted prevalence of T6SS-2 was very low in phylogroups F and G, a finding similar to what was previously reported in intestinal pathogenic *E. coli* (InPEC) and ExPEC, where the majority of genomes from F and G phylogroups presented either no T6SS or only T6SS-1, respectively (10). Altogether, these findings indicate that APEC strains from phylogroup G might have maintained the T6SS through evolution, and this contribution may therefore be one of the many factors that influence this phylogenetic divergence in APEC, resulting in the new T6SS phylogroup G.

Previous studies already proposed that T6SS in *E. coli* was probably acquired by HGT based on phylogenetic analysis using T6SS sequences from multiple bacterial species (8, 16). In our study, the majority of the T6SS clusters in ExPEC were associated with HGT elements, supporting this hypothesis. Additionally, the presence of accessory proteins between the 13 core protein clusters observed in the present study was in line with Boyer et al. (16), who associated the presence of accessory proteins with niche adaptation. Their study identified that bacterial species presenting the same T6SS protein profile were clustered together in the phylogenetic tree, and also shared the same environment, such as the ocean and plants (16). In the rice bacterial pathogen *Acidovorax avenae* subsp. *avenae*, the expression of T6SS genes *icmF, impB, impH,* and *duf877* was associated with changes in the environment, such as salt stress and low temperature, and with co-culture with another pathogen (49). In addition, the presence of accessory proteins in our T6SS-1 sequences represented the most variable region within this cluster, which consisted of DUF1240, LCAT, PGAP1, and lipase-2 proteins. This finding was in line with the findings from Shyntum and colleagues, who also found hypothetical proteins, including lipases and PAAR_motif, in the region between *tssA* and *tssF* in T6SS clusters from *Pantoea ananatis* (50).

Hence, we hypothesized that the presence of accessory proteins in the ExPEC genomes likely contributes to niche adaptation. In fact, a distinction between host and niche for T6SS-2 but not for T6SS-1 was observed in our study. Only genomes from NMEC strains isolated from cerebrospinal fluid were included in our analysis, to distinguish them from strains isolated from blood, which were considered as SEPEC, while for UPEC and APEC, only strains isolated from human urine and poultry colibacillosis cases were included, respectively. Our phylogenetic analysis based on T6SS-2 sequences indicates that the T6SS-2 cluster in ExPEC is likely pathotype- and consequently, niche-specific. This cluster might also be associated with high virulence traits in these pathotypes, since it was already described as overrepresented among pathogenic *E. coli* strains by Journet and Cascales (8), while the T6SS-1 cluster has been described to be involved in bacterial competition (8, 51, 52).

Our phylogenetic analyses also indicated that the presence of T6SS in NMEC is mostly associated with ST95 strains. ExPEC strains from the ST95 lineage usually exhibit a lower incidence of antimicrobial resistance, while ST131 strains are usually multidrug resistant (53, 54). This pattern was also observed in our study, since the majority of ST131 strains were UPEC and SEPEC, and had the highest prevalence of AMR genes, especially aminoglycosides, which was also observed among these two pathotypes. This suggests that in ExPEC, the presence of AMR genes is independent of T6SS. ExPEC strains possessing chromosomal T6SS typically do not carry multiple AMR genes, indicating that co-harboring both AMR genes and an active T6SS may not be advantageous for ExPEC strains and could potentially decrease their competitive fitness in specific niches. Our finding is in line with a recent study, which observed a mild negative association between T6SS and MDR across *E. coli* (10). Avoidance of co-occurrence between AMR genes and an active T6SS has also been observed in MDR *Acinetobacter baumanii* strains that regulate the expression of their T6SS through spontaneous plasmid loss mediated by repressors of the secretion system present in the plasmid (5).

In conclusion, this study highlights the distinct distribution and evolutionary dynamics of T6SS clusters among ExPEC pathotypes, underscoring their potential role in virulence and host/niche adaptation. Our findings also suggest that T6SS is a conserved virulence mechanism shared between NMEC and APEC. Notably, the emergence of phylogroup G as an APEC-specific lineage with genetic similarities to B2 strains implies a conservation of T6SS through species evolution. Additionally, our findings indicate an inverse relationship between T6SS presence and antimicrobial resistance. Since the study was limited to bioinformatic analysis, it was not possible to evaluate the functionality of T6SS, but to presume it. Despite its limitations, our study provides new insights into the presence of T6SS and particular ExPEC pathotypes, adaptation to niche/environment shaping the distribution of T6SS among ExPEC pathotypes and supports its relevance for further *in vitro* investigation regarding its expression and function in *Escherichia coli*.

## MATERIALS AND METHODS

### Whole genome sequencing and assembly

A total of 78 NMEC and 68 APEC genomes that belong to the Logue Lab collection were submitted for whole genome sequencing and assembly to Mr. DNA Molecular Research LP (Shallowater, TX). NMEC strains were isolated from the cerebrospinal fluid of infants with meningitis, as previously described (55, 56), whereas APEC strains were recovered from the lesions of poultry diagnosed with colibacillosis (57). Genomic DNA was extracted from each strain using the MagAttract HMW DNA Kit (Qiagen, Hilden, Germany). The DNA sample was quantified using Qubit dsDNA HS Assay Kit (Life Technologies, Carlsbad, CA), followed by analysis using E-Gel SizeSelect 2% Agarose Gel (Invitrogen, Waltham, MA). The samples were sheared using the Covaris G-tube (Covaris Inc.). The average size of the sheared DNA was determined using Agilent 2100 Bioanalyzer (Agilent Technologies). Then, 2,000–3,000 ng of the sheared and purified DNA was used as input for the library preparation using the SMRTbell Express Template Prep Kit 2.0 (Pacific Biosciences, Menlo Park, CA). The SMRT libraries were size selected (>6–7 bk) using 0.75% Agarose gel in BluePippin (Sage Science, Beverly, MA), followed by a DNA concentration check using the Qubit dsDNA HS Assays Kit (Life Technologies, Carlsbad, CA). Genomic sequencing was performed on the PacBio Sequel II (Pacific Biosciences). Raw reads were used for the assembly using HIFIASM v0.24.0 (58). Quality check on the assemblies was performed with QUAST v5.2.0 (59), and MultiQC v1.14 (60) was used to summarize the statistics. The resulting 146 ExPEC genomes were deposited in the National Center for Biotechnology Information (NCBI) under BioProject numbers PRJNA1383263 and PRJNA1395651. Accession numbers are included in Table S1.

### Genome collection

A total of 253 publicly available ExPEC genomes (NMEC = 67; UPEC = 90; APEC = 15; SEPEC = 81) were downloaded using the Bacterial and Viral Bioinformatics Resource Center (BV-BRC) platform, which combines data from the National Center for Biotechnology Information (NCBI) and the European Nucleotide Archive (ENA) databases. Keywords, such as *bacteremia/sepsis*, *urinary tract infection*, *cerebrospinal fluid*, and *colibacillosis*, were used as inclusion criteria when searching for *E. coli* genomes from the database, as well as host, genome quality, and completeness. All genomes analyzed in this study and their associated database accession numbers are listed in Table S1. QUAST v5.2.0 (59) was used to assess assembly quality using default parameters, and only genomes with less than 600 contigs and with an N50 above an arbitrary value of 14 kbp were included in the study.

### T6SS screening

The T6SS-1 and T6SS-2, used as templates for the identification of T6SS in the NMEC genomes, were obtained from the NMEC strains RS218 (NCBI acc. CP007149) and O18 (NCBI acc. CP007275), respectively. APEC T6SS-1, T6SS-2, and T6SS-3 sequences from the ED205 strain were used as templates in the screening of APEC genomes (GenBank acc. KF678353.1, KF678352.1, KF678351.1). Due to the lack of information on T6SS in UPEC and SEPEC, a subset of strains from the collection was analyzed with the T6SS prediction tool available on SecReT6 (https://bioinfo-mml.sjtu.edu.cn/SecReT6/index.php). The UPEC strain DS566 (NCBI acc. CP092534) and the SEPEC strain SF-468 (NCBI acc. CP012625) were identified as T6SS-positive, and their nucleotide sequences were subsequently used as templates for T6SS screening in the remaining genomes of their respective pathotypes. Nucleotide sequences of all T6SS references were submitted to Prodigal v2.6.3 (61) for protein-coding gene prediction and to obtain the amino acid translated sequence. The latter was further submitted to EggNOG-mapper v2.1.9 (62, 63) to identify orthologs and functional annotation. The analysis was able to identify the core genes, as well as genes that codify auto-immune proteins, and potential T6SS effectors, considered as accessory genes. The output files from both analyses were combined into a metadata table used as input for the linear visualization of each reference sequence using the “*gggenes*” package in RStudio v2025.05.0.496 (64) and colors were modified in PowerPoint.

All ExPEC genomes were aligned to their specific T6SS sequence templates using BLASTn (65). A Python script was developed to parse and filter hits longer than 500 bp and concatenate hits on the same genome, resulting in one single sequence for each genome and saving the output as FASTA file. The FASTA file was used as input to visualize the length of the sequences on Geneious v2024.0.5 (https://www.geneious.com), and the XML output file from BLASTn was manually curated. To establish the presence of a specific cluster, a query cover and percentage identity threshold of 52% and 70% were set, respectively. The positive sequences for T6SS were manually selected on Geneious v2024.0.5 and used as input for further analysis. Prodigal v2.6.3 (61) was used to obtain annotation files in GFF format from all T6SS nucleotide sequences and to translate them into amino acid sequences, resulting in an FAA file. The amino acid sequences were submitted to EggNOG-mapper v2.1.9 (62, 63) to obtain the protein identification within the cluster of each T6SS sequence. The protein family (Pfam) category was used as the main identifier for further analysis, followed by a confirmation using BLASTp. A Python script was developed to analyze and create a presence/absence matrix, which was used to identify the structure of each T6SS locus among the sequences, as well as the prevalence of each pattern. Additionally, the matrix was used as input to create a heatmap using Matplotlib (66). Sequences were considered incomplete if lacking ≥3 proteins, and complete if only two proteins are missing. All Python scripts were run locally and are available on https://github.com/IenesLimaJ/ExPEC_T6SS_paper.

### T6SS phylogenetic analysis

The T6SS nucleotide sequences from all pathotypes were aligned by locus using MAFFT v7.526 (67) available on Geneious v2024.0.5. FastTree v2.1.10 (68) was used to build a maximum-likelihood phylogenetic tree using the GTR + CAT nucleotide evolution model due to the number and unequal length of sequences. All remaining parameters were set as default. The phylogenetic trees were visualized and annotated on RStudio v2025.05.0.496 using the packages “*ggtree*” (69–72), “*ggstar*” (73), and “*treeio*” (74). Additionally, the MAFFT alignment also created a distance matrix based on the percentage identity between the nucleotide sequences of T6SS, which was used to create a clustered heatmap using the “*pheatmap*” (75) package on RStudio.

### ExPEC genomic characterization

The O- and H- antigen were identified using *ecoh* database available on ABRicate v.1.0.1 (76) or using SerotypeFinder (Center for Genomic Epidemiology Server, https://cge.food.dtu.dk/services/SerotypeFinder/) with an ID threshold and minimum length of 85% and 60%, respectively. The multi-locus sequence type of each strain was determined using the *ecoli_achtman_4* database available on MLST v.2.23.0 (Seemann T, mlst Github https://github.com/tseemann/mlst), which scans contig files against the PubMLST typing schemes (77) based on the seven house-keeping loci for *E. coli* (*adk*, *fumC*, *gyrB*, *icd*, *mdh*, *purA*, and *recA*). The result was used as input for the ST clonal complex identification, which was performed manually using “*Escherichia* typing” tool available on PubMLST (78), using the MLST (Achtman) scheme. EzClermont v.0.7.0 (https://github.com/nickp60/EzClermont) was used to identify the *E. coli* phylogenetic group. AMRFinderPlus v4.0.3 (79) was used for antimicrobial resistance genes screening, and based on the prevalence of antimicrobial class per strain, the isolates were categorized as *potential* single-resistant (SR), double-resistant (DR), and multidrug-resistant (MDR) using criteria described by Magiorakos et al. (80).

A 60% coverage and 70% identity cut-off were employed to determine a positive presence for each gene. Alien_hunter (https://www.sanger.ac.uk/tool/alien_hunter/) was used for identification of horizontal gene transfer (HGT) events. The reference genomes from each pathotype, their corresponding T6SS sequences, and the HGT coordinates obtained from Alien_hunter were uploaded to Proksee (https://proksee.ca/) for circular genome visualization. For detailed visualization, the location of all HGT regions was manually added to their corresponding reference genome on Geneious v2024.0.5, followed by the alignment of the T6SS locus to the genomes using MAFFT as previously described. All analyses mentioned above were performed using the Georgia Advanced Computing Resource Center (GACRC) at the University of Georgia, except for the SerotypeFinder, which was run on the Center for Genomic Epidemiology online server as mentioned above. Unless otherwise noted, default parameters were used for all software tools.

### Statistical analysis

To analyze the association between ExPEC variables (pathotypes, phylogroups, O-types, H-types, and ST-complexes) and the presence of T6SS loci, a logistic regression model was employed, using the “*glm*” function in RStudio v2024.09.1.394, which is suitable for binary outcome data (T6SS presence = 1; absence = 0) and allows the odds estimation of T6SS presence for each ExPEC variable. The “*emmeans*” package (81) was used to obtain the estimated marginal means and pairwise comparisons between certain variables, which were evaluated using Tukey-adjusted pairwise contrasts, and statistical significance was assessed with Wald z-tests. The association between multidrug resistance (MDR) and T6SS presence, contingency tables were constructed and analyzed using Pearson’s *χ* test. Because some categories contained low expected frequencies, Fisher’s exact test was also applied to ensure robustness of the association. To visualize differences between MDR and non-MDR genomes, a radar plot based on group-specific proportional distributions was generated using the “*ggradar*” package (https://github.com/ricardo-bion/ggradar) in RStudio v2025.09.2.

## Data Availability

The NMEC and APEC genomes sequenced in this study were submitted to NCBI under BioProject numbers PRJNA1383263 and PRJNA1395651, and the accession numbers are included in Table S1.

## SUPPLEMENTAL MATERIAL

Supplemental figures spectrum.04033-25-s0001.pdf Fig. S1 to S14.

Supplemental tables spectrum.04033-25-s0002.xlsx Tables S1 to S5.

ASM does not own the copyrights to Supplemental Material that may be linked to, or accessed through, an article. The authors have granted ASM a non-exclusive, world-wide license to publish the Supplemental Material files. Please contact the corresponding author directly for reuse.
