# Supplementary material for: Genomic characterization of the type VI secretion system in extraintestinal pathogenic Escherichia coli
Source: Microbiol Spectr. 2026 Jun 25;14(8):e04033-25. doi: 10.1128/spectrum.04033-25 (PMC13436143; doi:10.1128/spectrum.04033-25)
Supplement: Supplementary file 1 [file spectrum.04033-25-s0001.pdf]

SUPPLEMENTARY FIGURES

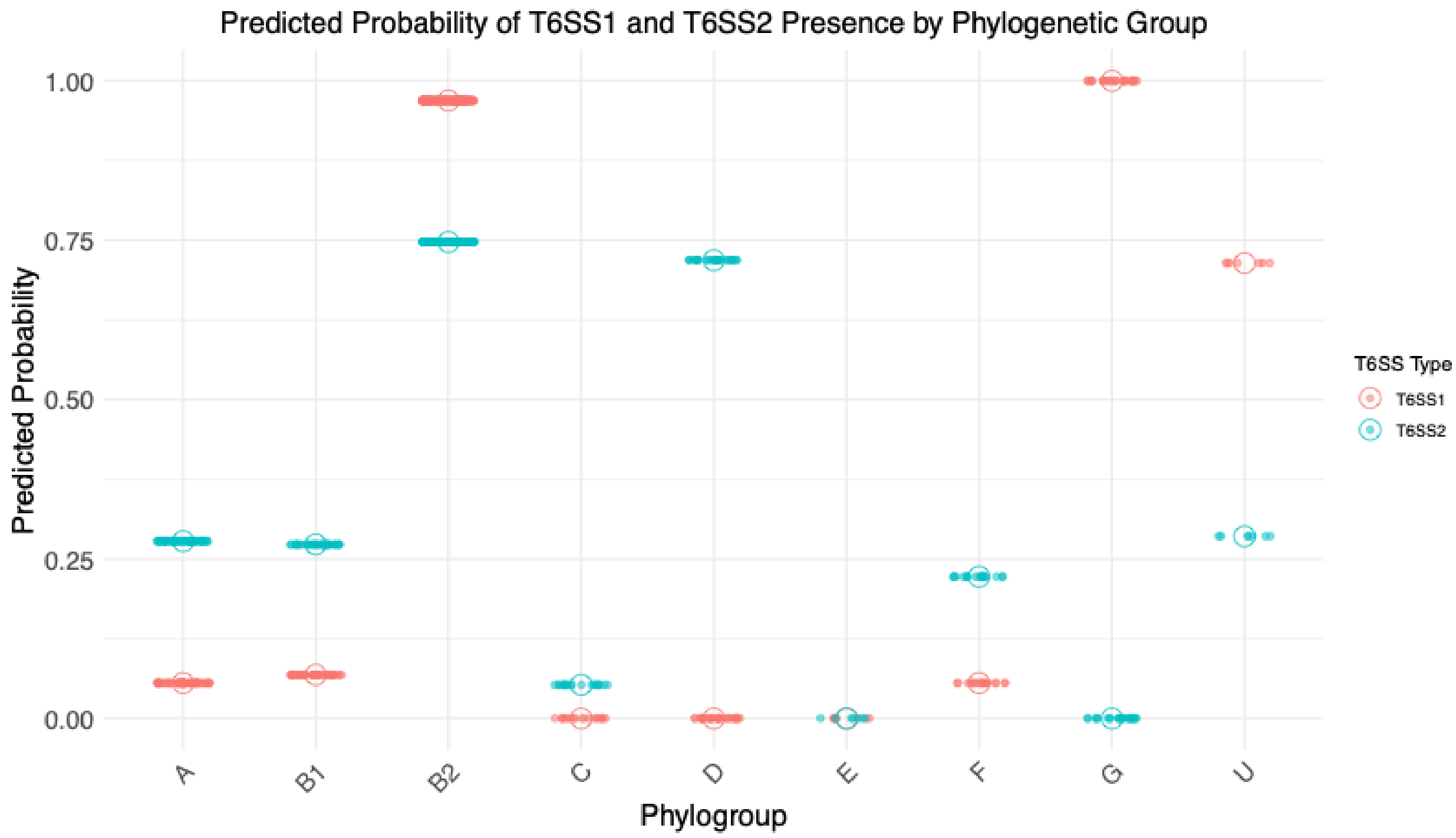

Supplementary Figure 1. Predicted probability of T6SS-1 and T6SS-2 presence by phylogenetic group of ExPEC.

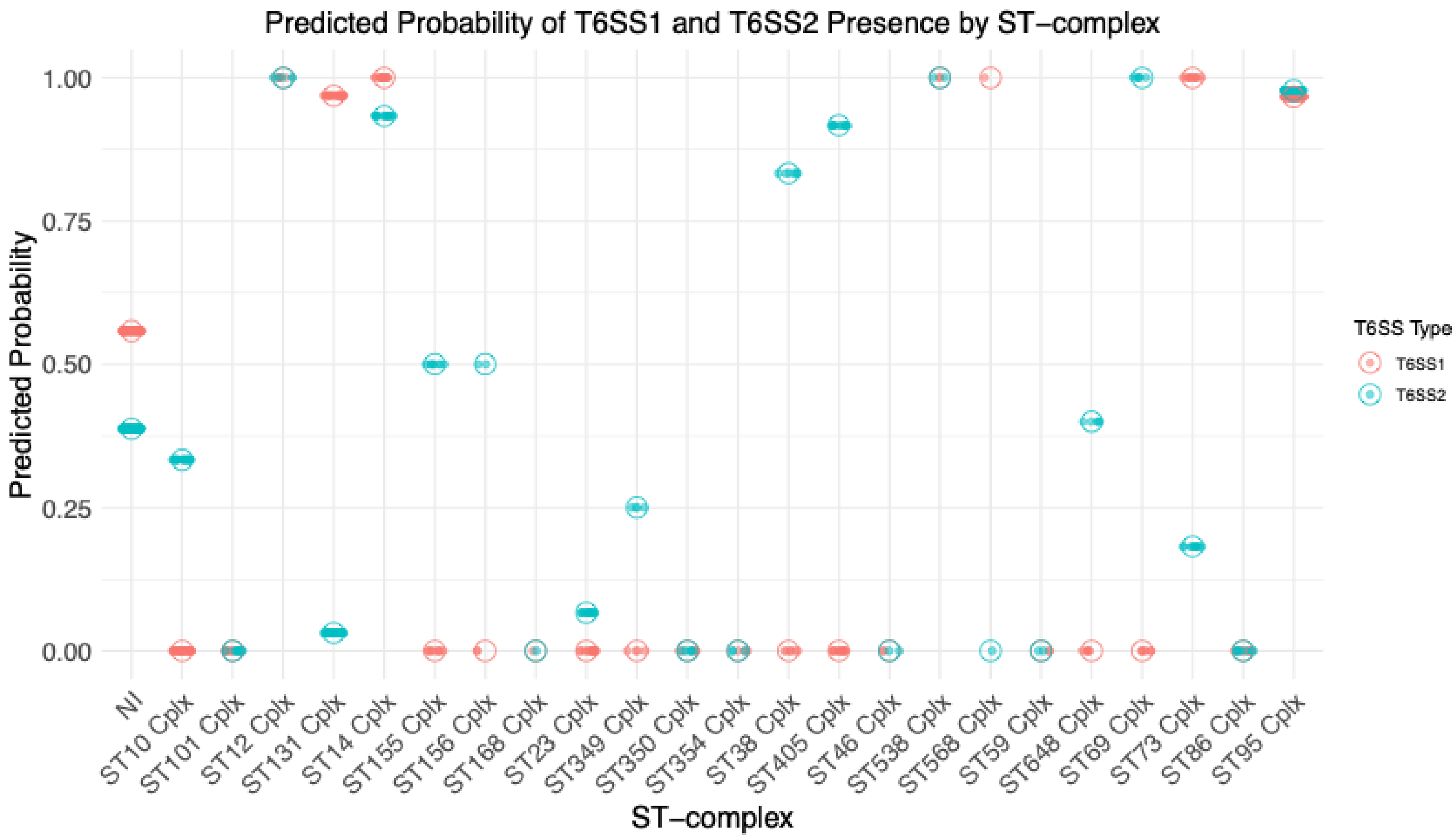

Supplementary Figure 2. Predicted probability of T6SS-1 and T6SS-2 by ST-complex of ExPEC.

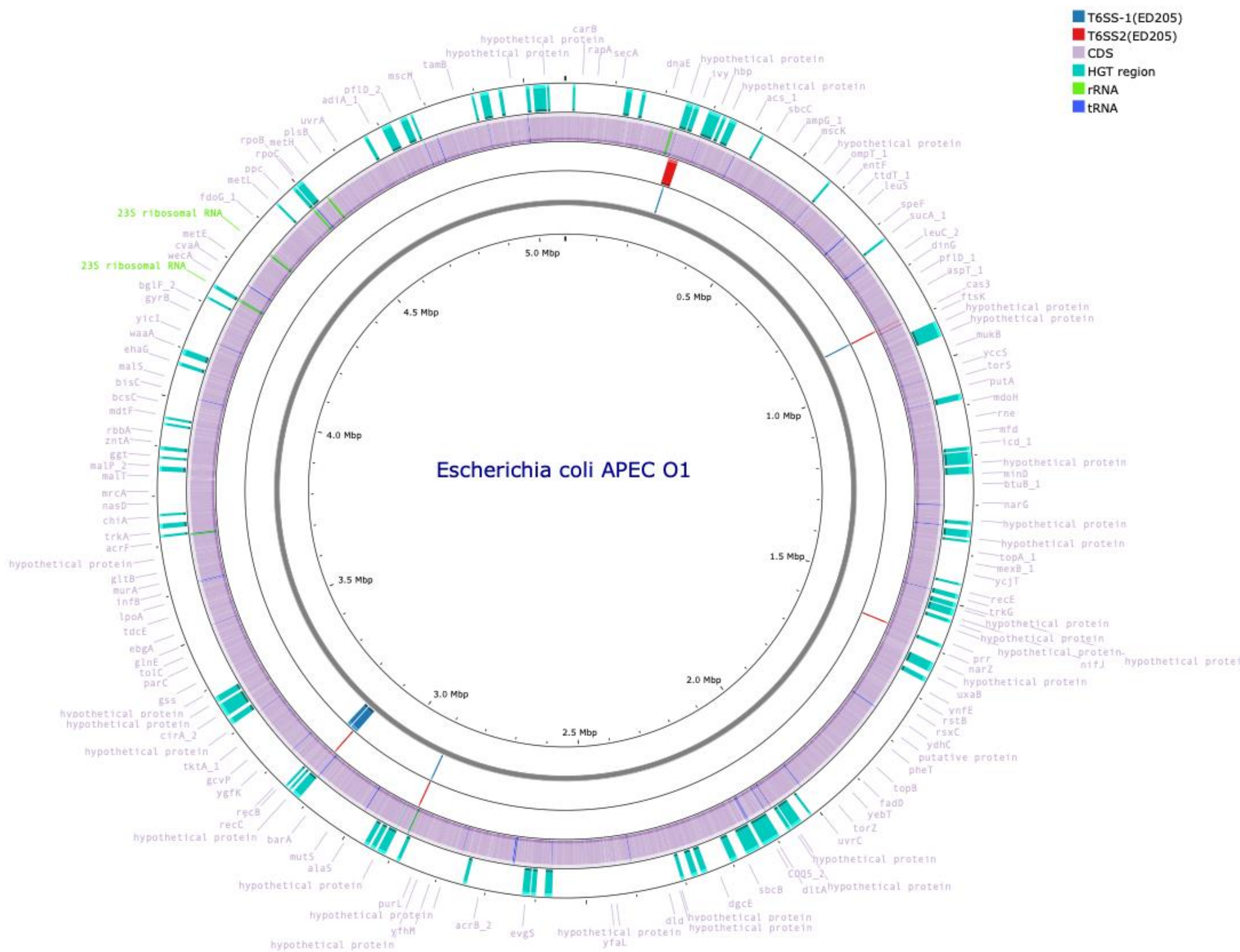

Supplementary Figure 3. Location of T6SS clusters and horizontal gene transfer (HGT) regions in the genome of the APEC strain APECO1. Gray backbone circle represents the full genome, whereas the following circles indicate the location of T6SS-1 (blue), T6SS-2 (red), the CDS regions (purple), and the outer circle indicates the HGT regions (teal).

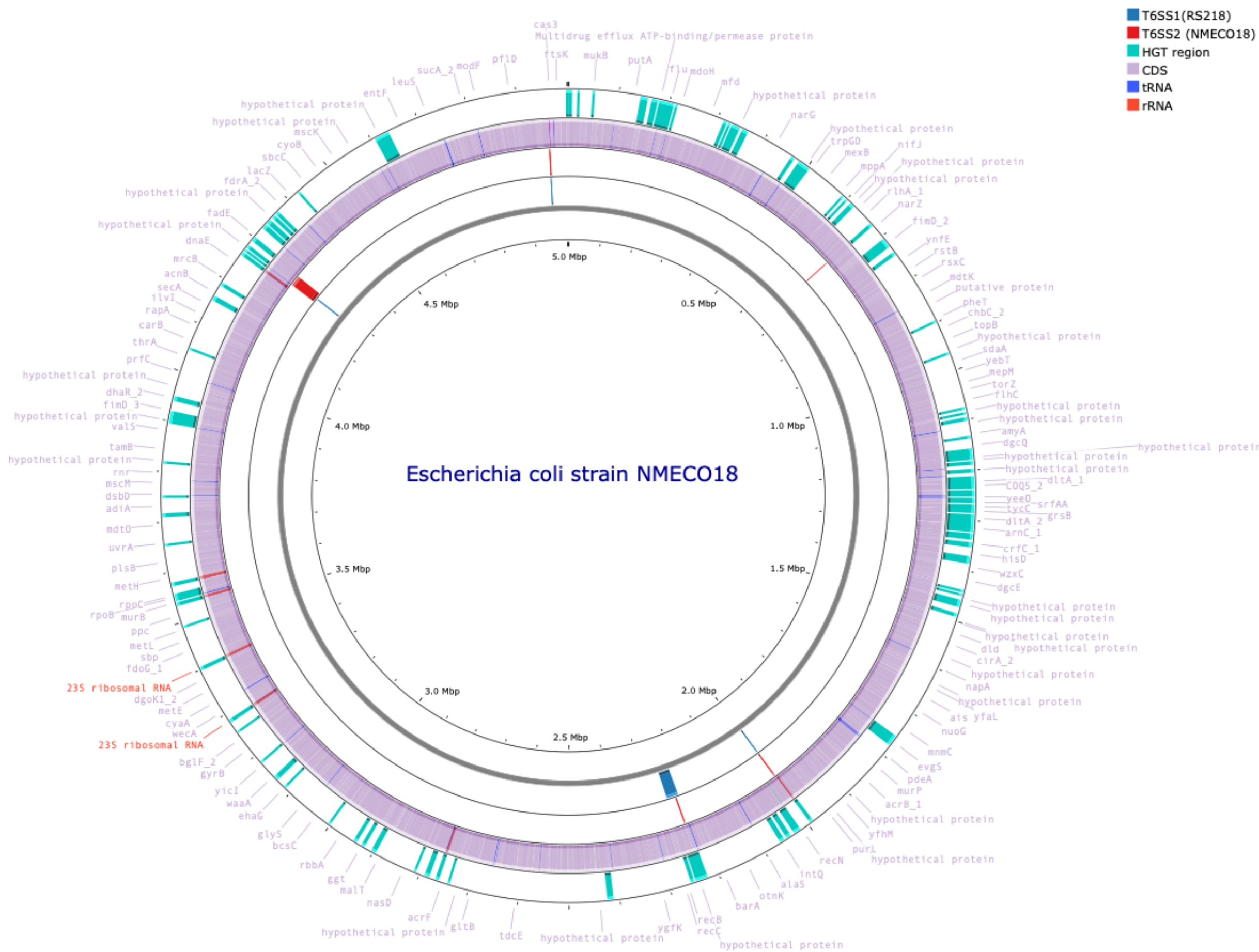

Supplementary Figure 4. Location of T6SS clusters and horizontal gene transfer (HGT) regions in the genome of the NMEC strain NMECO18. Gray backbone circle represents the full genome, whereas the following circles indicate the location of T6SS-1 (blue), T6SS-2 (red), the CDS regions (purple), and the outer circle indicates the HGT regions (teal).

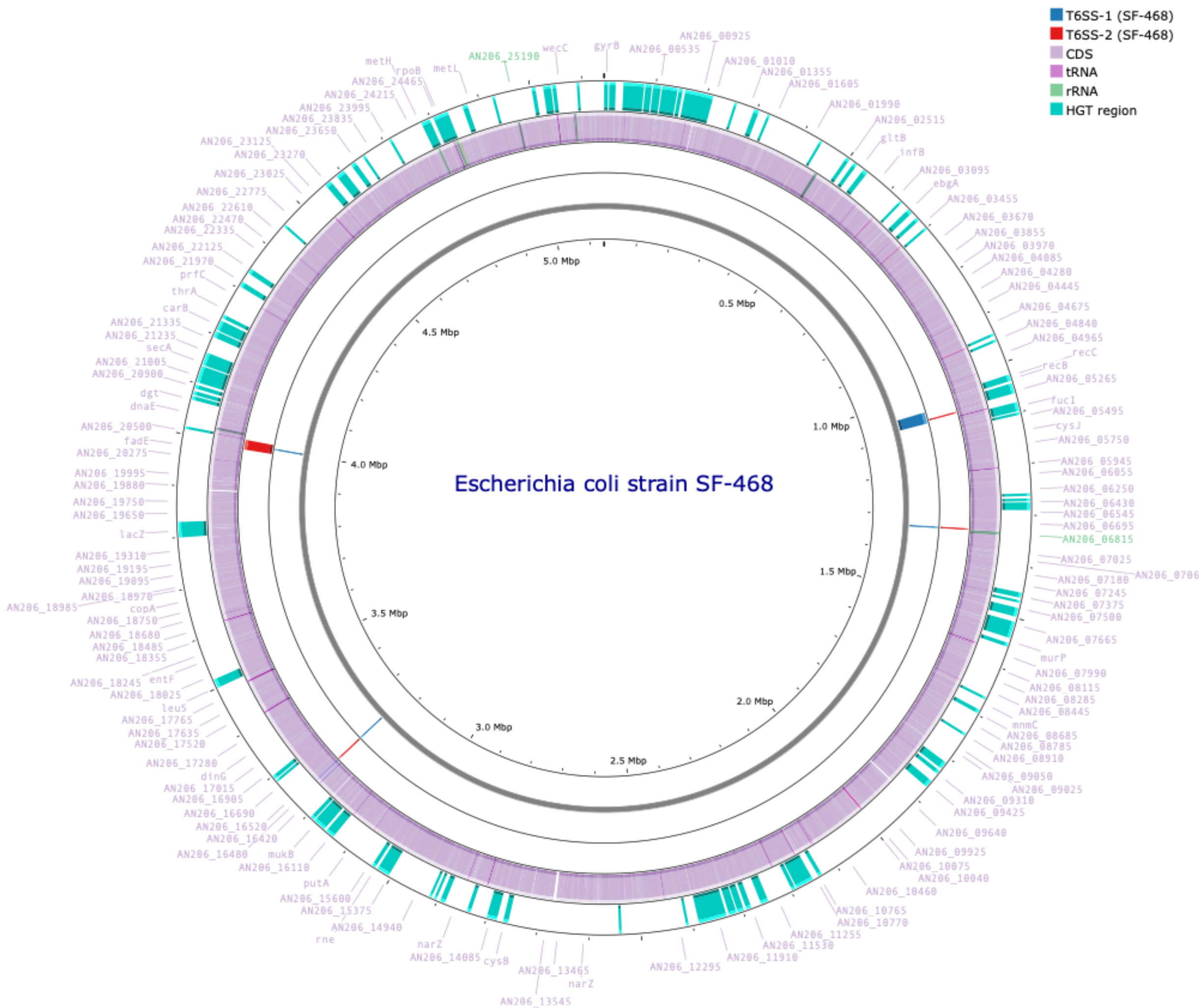

Supplementary Figure 5. Location of T6SS clusters and horizontal gene transfer (HGT) regions in the genome of the SEPEC strain SF-468. Gray backbone circle represents the full genome, whereas the following circles indicate the location of T6SS-1 (blue), T6SS-2 (red), the CDS regions (purple), and the outer circle indicates the HGT regions (teal).

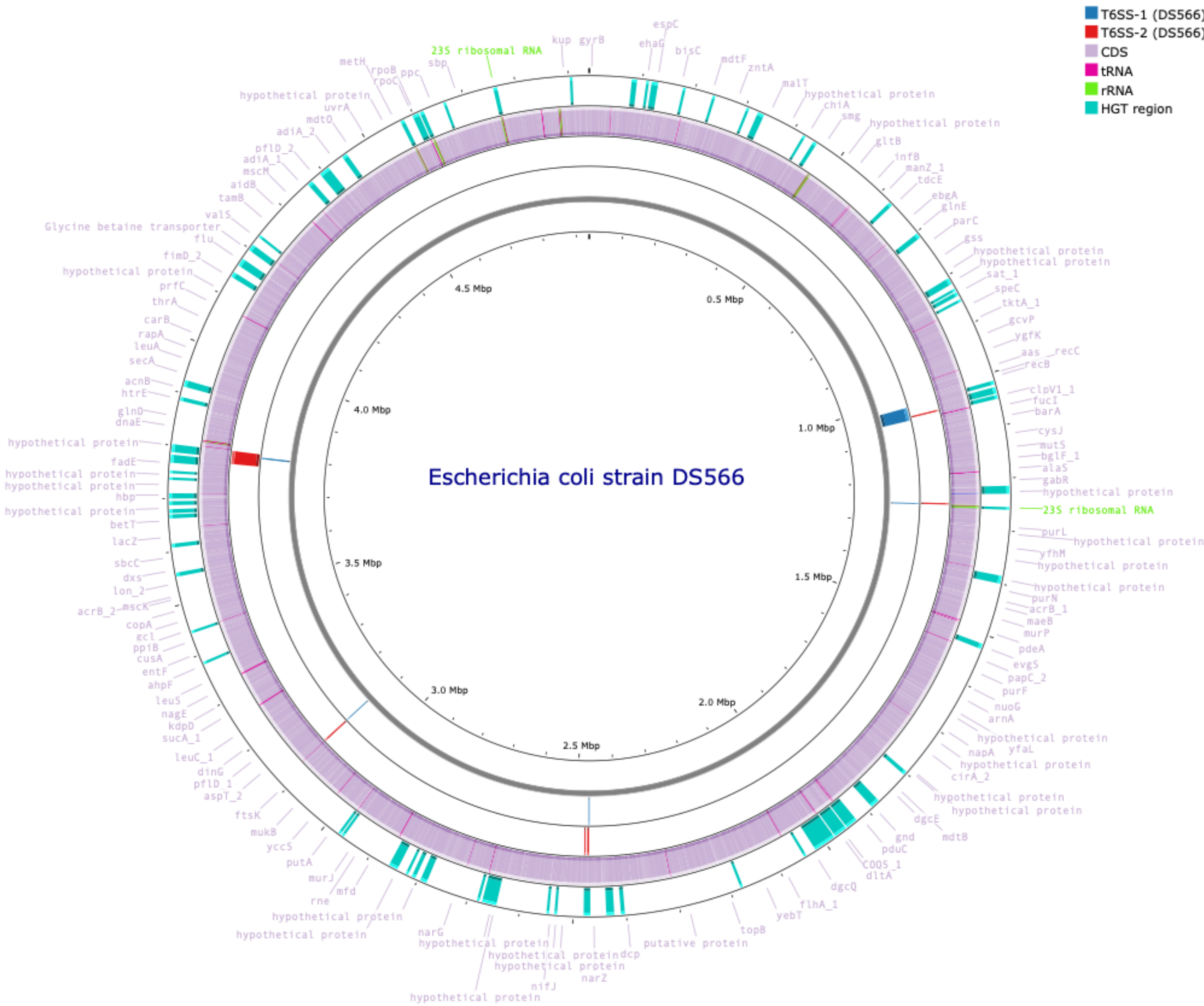

Supplementary Figure 6. Location of T6SS clusters and horizontal gene transfer (HGT) regions in the genome of the UPEC strain DS566. Gray backbone circle represents the full genome, whereas the following circles indicate the location of T6SS-1 (blue), T6SS-2 (red), the CDS regions (purple), and the outer circle indicates the HGT regions (teal).

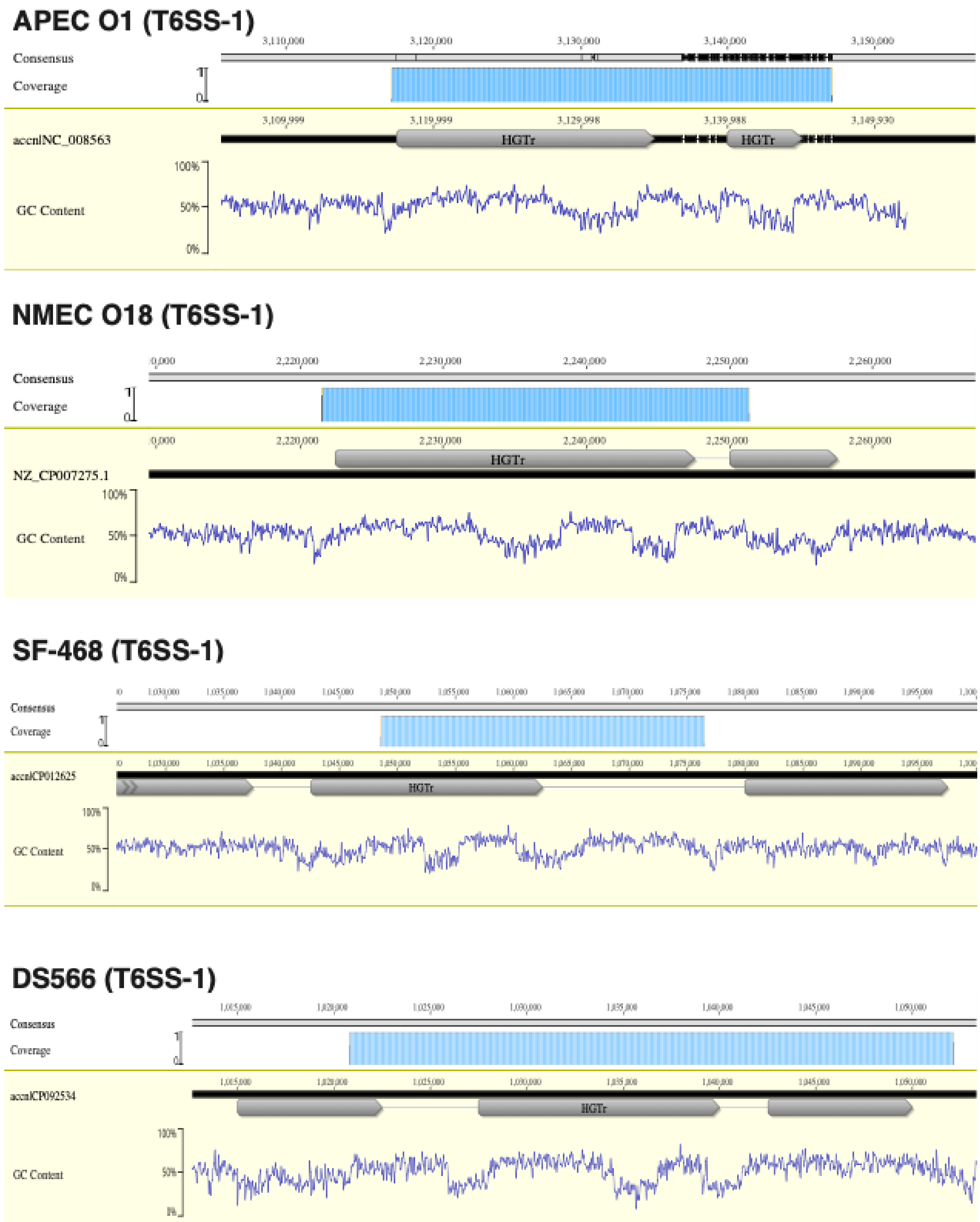

Supplementary Figure 7. Detailed location of T6SS-1 and horizontal gene transfer regions (gray arrows) in the ExPEC reference genomes.

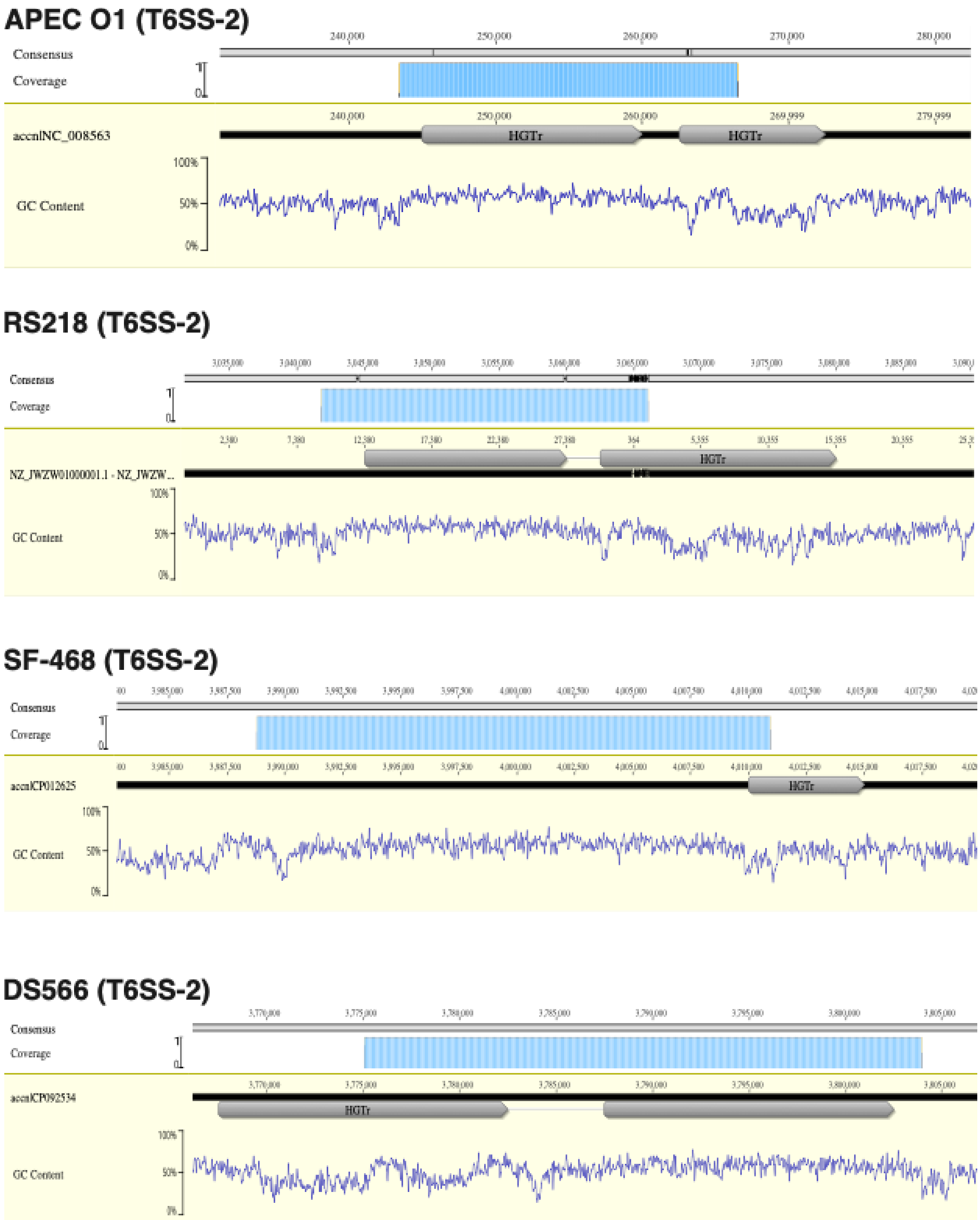

Supplementary Figure 8. Detailed location of T6SS-2 and horizontal gene transfer regions (gray arrows) in the ExPEC reference genomes.

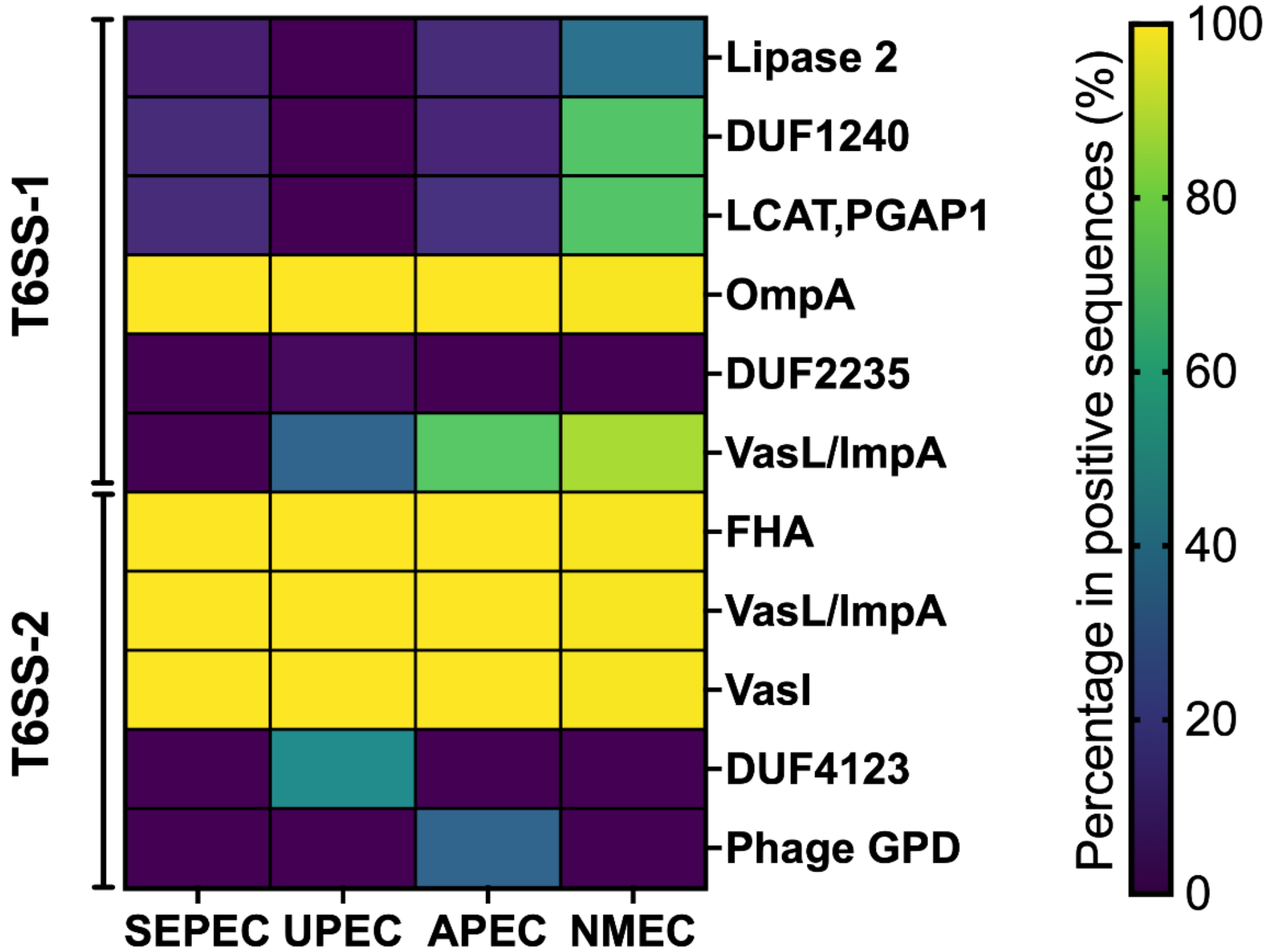

Supplementary Figure 9. Distribution of accessory proteins per T6SS cluster across the ExPEC pathotypes.

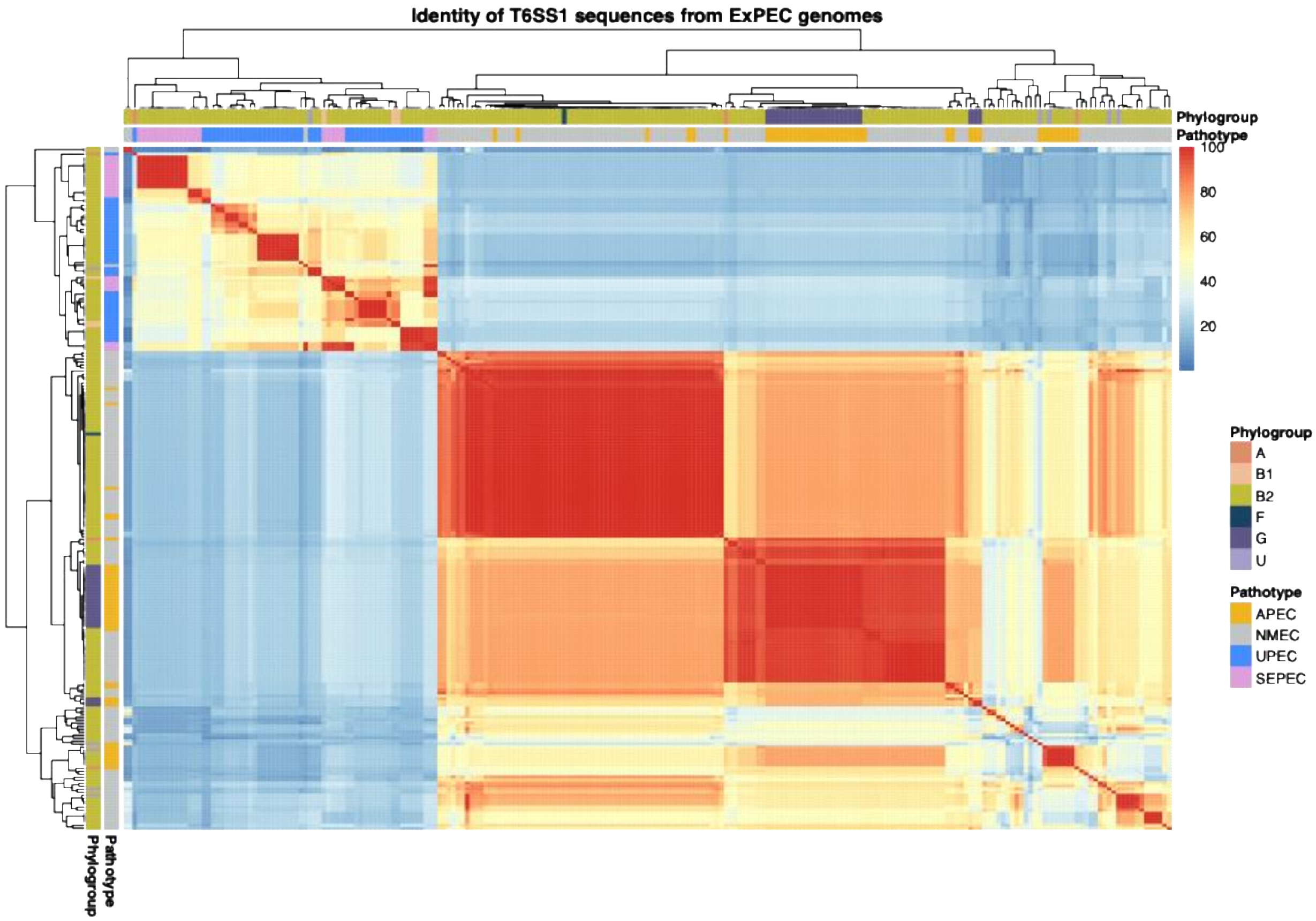

Supplementary Figure 10. Distance matrix using the nucleotide percentage identity between all T6SS-1 sequences of ExPEC obtained from MAFFT alignments. Heatmap created using *pheatmap* on RStudio.

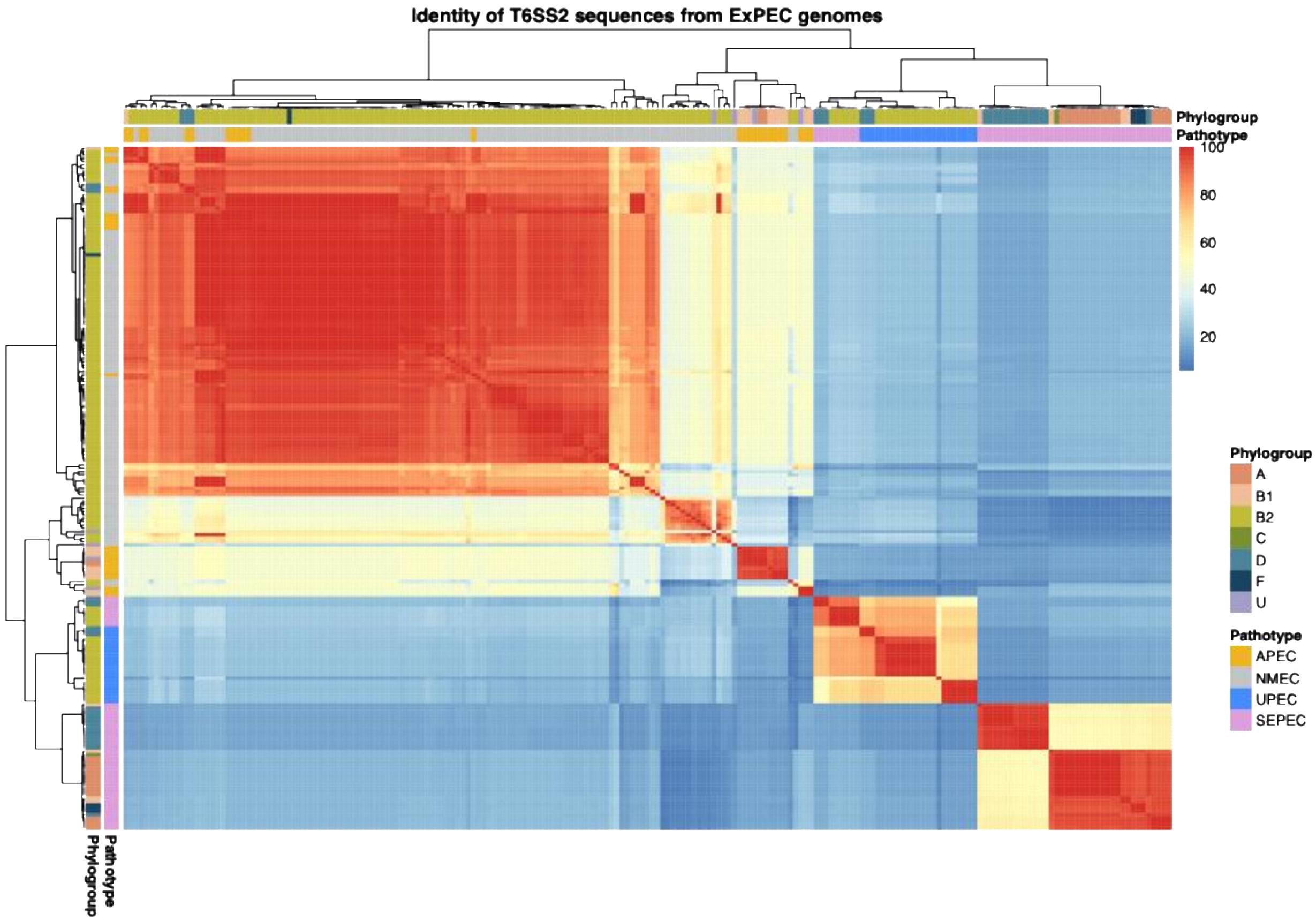

Supplementary Figure 11. Distance matrix using the nucleotide percentage identity between all T6SS-2 sequences of ExPEC obtained from MAFFT alignments. Heatmap created using *pheatmap* on RStudio.

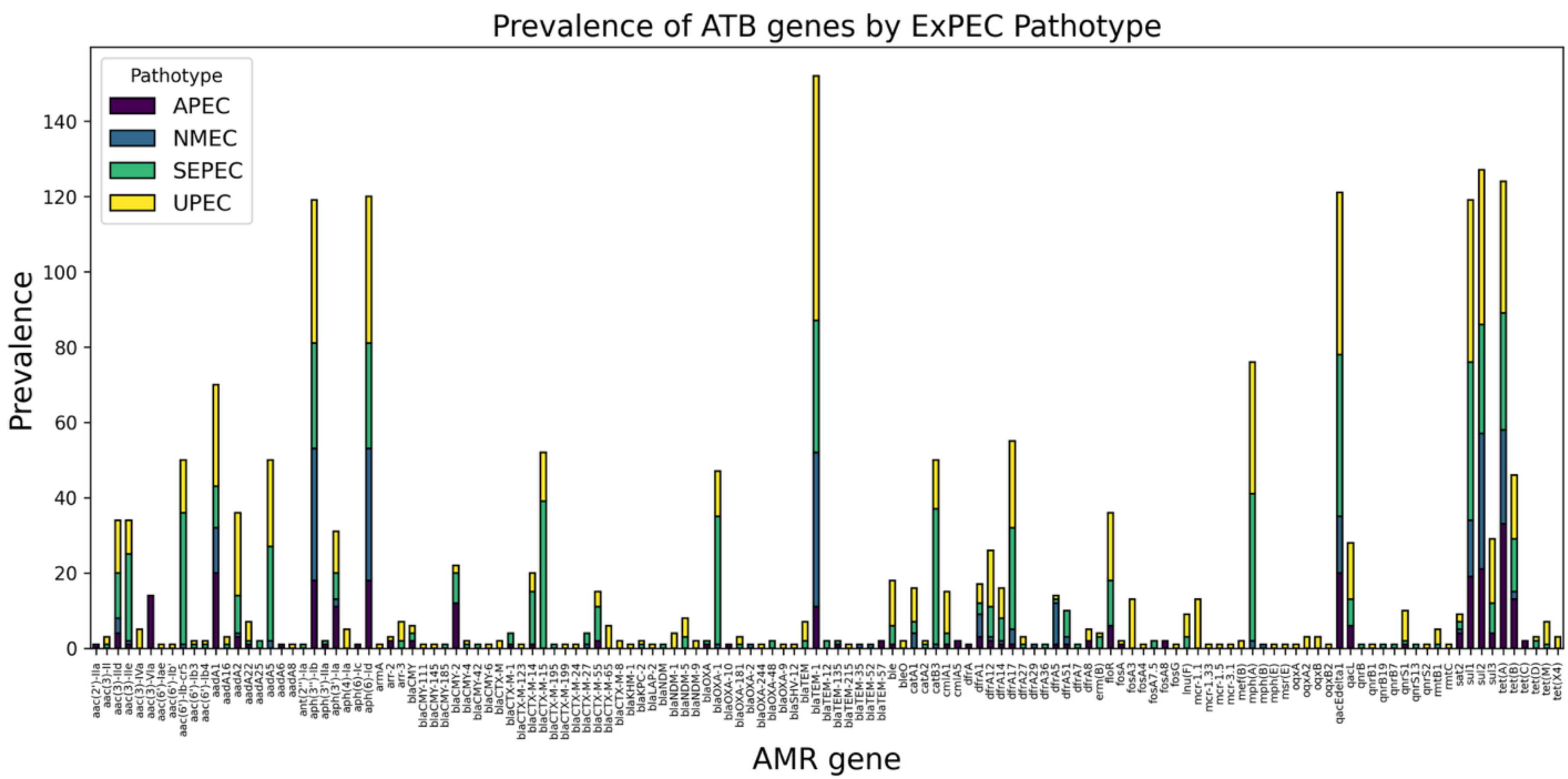

Prevalence of ATB genes by ExPEC Pathotype
Pathotype
APEC
NMEC
SEPEC
UPEC
Prevalence
0
20
40
60
80
100
120
140
AMR gene
aac(2')-IIa
aac(3)-II
aac(3)-IId
aac(3)-IIe
aac(3)-IVa
aac(3)-VIa
aac(6')-Iae
aac(6')-Ib'
aac(6')-Ib-cr5
aac(6')-Ib3
aac(6')-Ib4
aadA1
aadA16
aadA2
aadA22
aadA25
aadA5
aadA6
aadA8
ant(2'')-Ia
aph(3'')-Ib
aph(3')-IIa
aph(3')-Ia
aph(4)-Ia
aph(6)-Ic
aph(6)-Id
armA
arr-2
arr-3
blaCMY
blaCMY-111
blaCMY-145
blaCMY-185
blaCMY-2
blaCMY-4
blaCMY-42
blaCMY-6
blaCTX-M
blaCTX-M-1
blaCTX-M-123
blaCTX-M-14
blaCTX-M-15
blaCTX-M-195
blaCTX-M-199
blaCTX-M-24
blaCTX-M-27
blaCTX-M-55
blaCTX-M-65
blaCTX-M-8
blaKHM-1
blaKPC-2
blaLAP-2
blaNDM
blaNDM-1
blaNDM-5
blaNDM-9
blaOXA
blaOXA-1
blaOXA-10
blaOXA-181
blaOXA-2
blaOXA-244
blaOXA-48
blaOXA-9
blaSHV-12
blaTEM
blaTEM-1
blaTEM-12
blaTEM-135
blaTEM-215
blaTEM-35
blaTEM-52
blaTEM-57
ble
bleO
catA1
catA2
catB3
cmlA1
cmlA5
dfrA
dfrA1
dfrA12
dfrA14
dfrA17
dfrA27
dfrA29
dfrA36
dfrA5
dfrA51
dfrA7
dfrA8
erm(B)
floR
fosA
fosA3
fosA4
fosA7.5
fosA8
fosG
lnu(F)
mcr-1.1
mcr-1.33
mcr-1.5
mcr-3.1
mef(B)
mph(A)
mph(B)
mph(E)
msr(E)
oqxA
oqxA2
oqxB
oqxB2
qacEdelta1
qacL
qnrB
qnrB1
qnrB19
qnrB7
qnrS1
qnrS13
qnrS2
rmtB1
rmtC
sat2
sul1
sul2
sul3
tet(A)
tet(B)
tet(C)
tet(D)
tet(M)
tet(X4)

Supplementary Figure 12. Prevalence of AMR genes among ExPEC genomes.

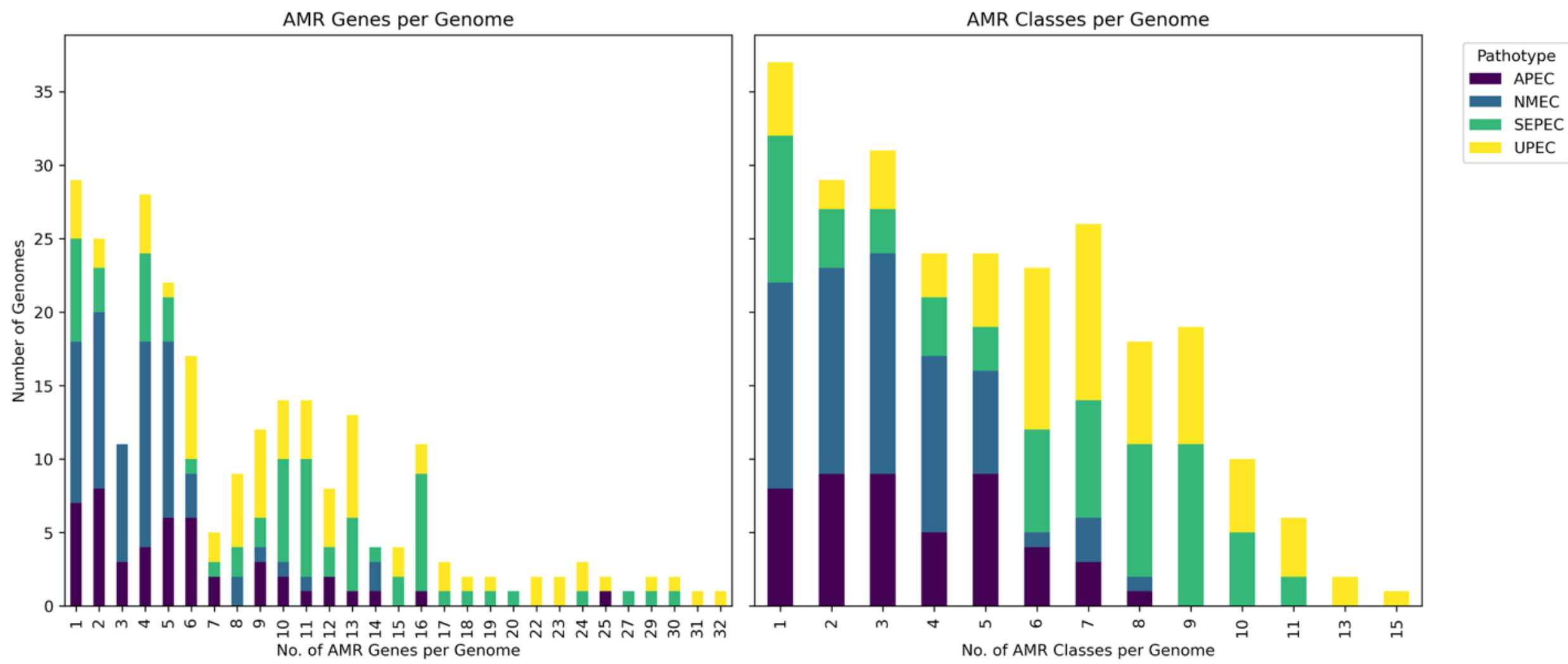

Supplementary Figure 13. Distribution of AMR genes (left) and classes (right) per genome among ExPEC.

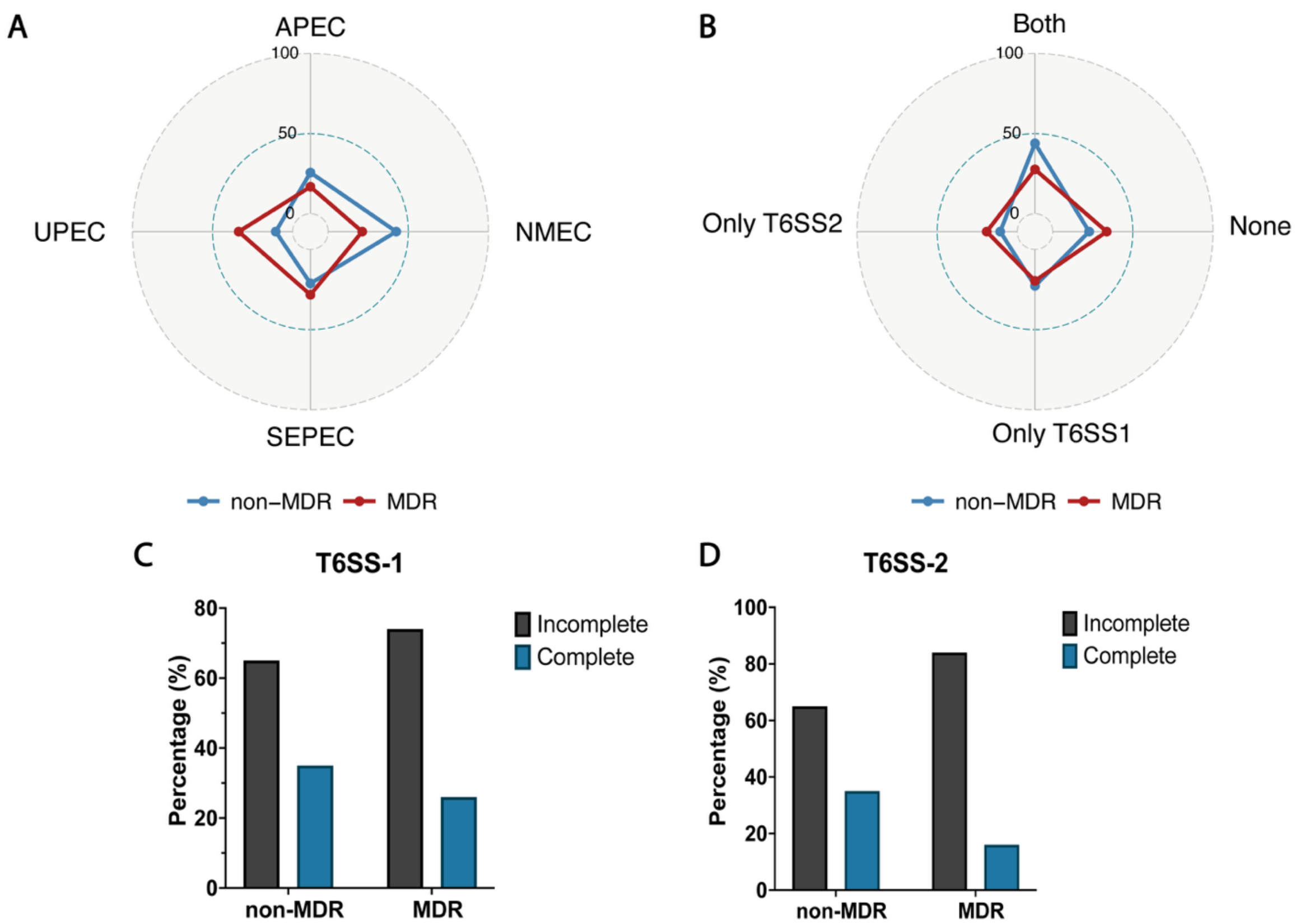

Supplementary Figure 14. Relative frequencies of multidrug resistance (MDR) between ExPEC pathotypes **(A,** $\chi^2 = 20.474$, df = 3, $p = 0.00013$; Fisher's exact test: $p = 0.0001$**)** and T6SS presence **(B,** $\chi^2 = 8.178$, df = 3, $p = 0.042$; Fisher's exact test: $p = 0.045$**)** in all ExPEC genomes analyzed (n = 399). Associations between MDR and T6SS cluster completeness were also investigated. **C)** Relative frequency of MDR and T6SS-1 completeness (Pearson's $\chi^2 = 2.086$, df = 1, $p = 0.148$; Fisher's exact test: $p = 0.154$). **D)** Relative frequency of MDR and T6SS-2 completeness (Pearson's $\chi^2 = 9.999$, df = 1, $p = 0.0016$; Fisher's exact test: $p = 0.0026$).
